# Evaluating modified diets and dietary supplement therapies for reducing muscle lipid accumulation and improving muscle function in neurofibromatosis type 1 (NF1)

**DOI:** 10.1371/journal.pone.0237097

**Published:** 2020-08-10

**Authors:** Emily R. Vasiljevski, Peter J. Houweling, Thusitha Rupasinghe, Tarneet Kaur, Matthew A. Summers, Ute Roessner, David G. Little, Aaron Schindeler

**Affiliations:** 1 Orthopaedic Research & Biotechnology Unit, The Children’s Hospital at Westmead, Westmead, New South Wales, Australia; 2 Discipline of Child & Adolescent Heath, Faculty of Medicine and Health, University of Sydney, Camperdown, New South Wales, Australia; 3 Murdoch Children’s Research Institute, Parkville, Victoria, Australia; 4 School of Biosciences, The University of Melbourne, Parkville, Victoria, Australia; 5 Healthy Ageing Theme, The Garvan Institute of Medical Research, Darlinghurst, New South Wales, Australia; 6 St Vincent’s Clinical School, Faculty of Medicine, University of New South Wales, Sydney, New South Wales, Australia; International Centre for Genetic Engineering and Biotechnology, ITALY

## Abstract

Neurofibromatosis type 1 (NF1) is a genetic disorder that affects a range of tissue systems, however the associated muscle weakness and fatigability can have a profound impact on quality of life. Prior studies using the limb-specific *Nf1* knockout mouse (*Nf1*_*Prx1*_^*-/-*^) revealed an accumulation of intramyocellular lipid (IMCL) that could be rescued by a diet supplemented with L-carnitine and enriched for medium-chain fatty acids (MCFAs). In this study we used the *Nf1*_*Prx1*_^*-/-*^ mouse to model a range of dietary interventions designed to reduce IMCL accumulation, and analyze using other modalities including *in situ* muscle physiology and lipid mass spectrometry. Histological IMCL accumulation was significantly reduced by a range of treatments including L-carnitine and high MCFAs alone. A low-fat diet did not affect IMCL, but did provide improvements to muscle strength. Supplementation yielded rapid improvements in IMCL within 4 weeks, but were lost once treatment was discontinued. *In situ* muscle measurements were highly variable in *Nf1*_*Prx1*_^*-/-*^ mice, attributable to the severe phenotype present in this model, with fusion of the hips and an overall small hind limb muscle size. Lipidome analysis enabled segregation of the normal and modified chow diets, and fatty acid data suggested increased muscle lipolysis with the intervention. Acylcarnitines were also affected, suggestive of a mitochondrial fatty acid oxidation disorder. These data support the theory that NF1 is a lipid storage disease that can be treated by dietary intervention, and encourages future human trials.

## Introduction

Neurofibromatosis type 1 (NF1) is a complex genetic disease that can have a profound impact on childhood development and adult quality of life. A clinical diagnosis of NF1 relies on fulfilling at least two of the seven diagnostic criteria; café au lait macules, skinfold freckling, neurofibromas, Lisch nodules, optic pathway tumors, bone dysplasia, and a family history [1]. Individuals with NF1 are predisposed to neural tumor formation and development of neurological, musculoskeletal and vascular abnormalities that contribute to the morbidity of the disorder. However, the reduced muscle tone, muscle weakness, poor co-ordination, and increased fatigability associated with NF1 are being increasingly appreciated as major burdens of disease [2]. These can lead to significant functional impairment and reduced quality of life in children, particularly when combined with other features of NF1 such as learning and behavioral difficulties [3].

Key insights into the role of *NF1* in muscle have come from conditional *Nf1*-deficient mouse lines. A limb-specific *Nf1* knockout mouse (*Nf1*_*Prx1*_^*-/-*^) was generated using a *Prx1*-cre transgene to drive deletion of *Nf1* in cells of the mesenchymal lineage in the fore and hind limbs. This mouse strain has reduced muscle weight, muscle weakness, fibrosis and impaired myoblast differentiation in the developing limbs [4]. To more specifically investigate the function of *Nf1* in muscle, Sullivan *et al*. generated a *Nf1* knockout mouse specifically deleting the gene in skeletal muscle (*Nf1*_*MyoD*_^*-/-*^) [5]. *Nf1*_*MyoD*_^*-/-*^ pups are born with a reduced body weight, exhibit stunted growth and failure to thrive, and maternal infanticide typically occurs during the first week of life. Electron microscopy analysis of *Nf1*_*MyoD*_^*-/-*^ muscle revealed excessive accumulations of intramyocellular lipid (IMCL), consistent with a metabolic myopathy [5]. This was confirmed at the light microscopy level by Oil Red O staining. These novel findings led us to speculate that *NF1* may have a key role in the regulation of muscle lipid metabolism.

More recently, Summers et al. published a report examining the IMCL found in *Nf1*_*MyoD*_^*-/-*^ muscle [6]. Lipidomics identified an increase in triglycerides, diglycerides, and cholesterol esters containing long-chain fatty acids (LCFAs). This led to the hypothesis that a deficiency in LCFA metabolism may underlie the muscle weakness. Consequently, a dietary intervention where *Nf1*_*Prx1*_^*-/-*^ mice were treated with a diet enriched for medium-chain fatty acids (MCFA) and supplemented with 300mg/kg L-carnitine led to reversal of the muscle lipid phenotype and improved forelimb grip strength. L-carnitine has previously been shown to treat muscle weakness in patients with other metabolic myopathies [7–9].

However, the mouse chow containing 70% octanoic acid as a MCFA source [6] is an approach that is not directly translational to dietary modification in humans. Moreover, it was unknown whether L-carnitine supplementation alone would be sufficient to produce significant reductions in muscle lipid and improvements in strength; an intervention that could be more readily adopted than changes in dietary fat intake. Thus the aim of this study was to use the published preclinical model to guide future clinical trials in individuals with NF1.

In addition to positive control (70% FAs ≤C12:0, 300mg/kg/day L-carnitine) and negative control (standard chow) groups, a number of other treatments were tested. These included a mitochondrial cocktail of L-carnitine, CoQ10, riboflavin (VitB2) and creatine [10]. This combination nutraceutical therapy can target many pathways of cellular energy dysfunction, including mitochondrial energy depletion and oxidative stress, which can be amendable by this approach in several lipid storage myopathies (LSM) [11]. We further included a group modelling limited dietary compliance, being fed normal chow 2 days per week. Finally, we tested mice given a low fat (<2%) diet, to see whether a generic low-fat strategy is effective.

The primary outcome measure for this study was IMCL accumulation, as measured by Oil Red O staining, and detailed in some cases by lipidomics. Our prior report had shown variability with grip strength testing in *Nf1*_*Prx1*_^*-/-*^ mice. Thus, *in situ* muscle physiology testing was performed to better functionally assess the effects of dietary interventions on hind limb muscle strength and fatigability.

### Study design

The screening of dietary strategies and dietary supplements (Table 1) was performed over 8 weeks, as previously shown to produce a significant decrease in IMCL in group 2 compared to group 1 [6]. Outcome measures included functional and histological assessments (n = 10/group). Tibialis anterior (TA) muscle was measured for maximum specific muscle force (mN/mm^2^), as well as the rate of muscle fatigue and recovery [12–14]. Hind limb wet muscle weight was also measured and frozen mouse quadriceps muscle, with other muscle groups (soleus, TA, and gastrocnemius) collected for comparison via Oil Red O staining for neutral lipids.

**Table 1:** Screening of dietary strategies and dietary supplements to reduce muscle lipid accumulation and improve muscle function.

Group	Diet	N
1	Standard chow	10
2	High MCFA chow + L-carnitine	10
3	High MCFA chow + L-carnitine (5/7d) Standard chow (2/7d)	10
4	High MCFA chow (no L-carnitine)	10
5	Standard chow + L-carnitine	10
6	Standard chow + mitochondrial cocktail	10
7	Low fat chow	10

In this subsequent study, High MCFA chow + L-carnitine was tested in a longitudinal study to assess the onset of reduced IMCL as well as the effects of longer-term treatment (Table 2). A third group was also tested, which was reverted to standard chow at week 8 to examine the potential restoration of IMCL with an unrestricted diet. Outcome measures for this study were histological staining (Oil Red O, n = 6 per group per time point) as well as a more detailed lipid analysis by LC-MS/MS and GC-MS/MS mass spectrometry of the week 8 time point (n = 8 per group).

**Table 2:** Timing and resilience of therapeutic treatment.

Group	Genotype	Diet	N	Time Points
1	WT	Standard chow	8	Weeks 8
2	Nf1_Prx1_^-/-^	Standard chow	26	Weeks 4, 8, 16
3	Nf1_Prx1_^-/-^	Modified chow	26	Weeks 4, 8, 16
4	Nf1_Prx1_^-/-^	Modified chow, standard chow (w8-16)	12	Weeks 12, 16

The original registered report protocol can be found at https://osf.io/mjc8u.

## Materials and methods

### Mouse strains and husbandry

All animal experiments were approved by the Westmead Hospital Animal Ethics Committee, The Children’s Hospital at Westmead/Children’s Medical Research Institute Animal Ethics Committee (protocol number: K319) or Murdoch Children’s Research Institute Animal Ethics Committee (protocol number: A879), and performed according to their prescribed guidelines. *Prx1-Cre* transgenic mice [15] and *Nf1*^*flox/flox*^ mice [16] (sourced from Jackson laboratory USA) were crossed to produce first generation *Prx1-Cre*^*+/-*^ *Nf1*^*flox/+*^ mice. They were then backcrossed to the parental *Nf1*^*flox/flox*^ strain to generate experimental homozygous knockout animals *Prx1 Cre*^*+/-*^ *Nf1*^*flox/flox*^.

*Nf1*_*Prx1*_^*-/-*^ mice were distinctly smaller than their littermates. To ensure their survival and reduce maternal rejection, pups were given daily saline injections of 0.1 mL up until four weeks of age. Samples were collected at three weeks of age for genotyping by quantitative real-time PCR for the *Cre* and *Nf1*^*flox*^ alleles (Transnet YX, TN, USA). All *Nf1*_*Prx1*_^*-/-*^ mice used in this study were age matched females.

All experimental animals were monitored twice daily throughout the course of the study. If any mice showed signs of distress or deterioration, or greater than >10% weight loss then 0.1mL saline injections were administered daily until weight normalized. Humane endpoints were defined as >10% weight loss or significant signs of distress that persisted. Mice were anesthetized by isofluorane inhalation prior to cardiac puncture and euthanasia by cervical dislocation after the completion of studies.

### Modified diets and dietary supplement therapies

Female mice were grouped housed 3–5 mice per cage, and fed *ad libitum* either standard AIN93M rodent chow pellets, or were assigned one of the modified diet chows. Those on the intermittent feeding regimens received 5 days of modified chow followed by 2 days of standard chow. All modified diet formulas were based on AIN93M and were designed to contain equal amounts of digestible energy (15.7 MJ/kg), carbohydrates (65.6–65.8%), protein (13.8–13.9%) and total fats (4%), excluding the low fat chow, which contained 15.0 MJ/Kg of digestible energy, a higher amount of carbohydrates (68.6%), and minimal requirements for total fats (1.8%).

High MCFA chow contains octanoic acid (C8:0, 2.8%) as its predominant lipid source, representing 70% of the total fatty acids content. LCFAs were included at minimal levels for animal health (Palmitic Acid 16:0, 0.07%; Stearic Acid 18:0, 0.03%; Oleic Acid 18:1, 0.17%; Linoleic Acid 18:2 n6, 0.61%; Linolenic Acid 18:3 n3, 0.30%). In contrast, the standard AIN93M, contains 100% of fatty acids as ≥C16:0. Palmitic Acid 16:0, 0.17%; Stearic Acid 18:0, 0.08%; Oleic Acid 18:1, 2.22%; Gadoleic Acid 20:1, 0.04%; Linoleic Acid 18:2 n6, 0.86%; Linolenic Acid 18:3 n3, 0.56%). The low fat chow was based on the AIN93M diet but had reduced fat (<2%).

L-carnitine was added to standard or high MCFA chow, at a concentration of 1.71g/kg, achieving a desired daily dose of 300mg/kg/mouse/day. The mitochondrial cocktail chow consisted of L-carnitine added at 1.71g/kg (300mg/kg/mouse/day), CoQ10 added at 0.114g/kg (20mg/kg/mouse/day), creatine added at 0.057g/kg (10mg/kg/mouse/day) and riboflavin (active vitamin B2) added at 0.0684g/kg (12mg/kg/mouse/day). All additives were based on standard chow consumption rates (Specialty Feeds, WA, Australia).

### *In situ* muscle physiology

After 8 weeks of treatment *in situ* assessment of the TA was performed using the 1300A Whole Mouse Test System and 701C stimulator (Aurora Scientific). The mice were anaesthetized using isofluorane inhalation and placed on a heated platform (37°C) for the procedure. Briefly, a small incision was made in the distal end of the animal’s leg and the skin is retracted halfway up the leg to expose the TA. The distal tendon of the TA was surgically isolated and the knee joint exposed. Surgical silk was used to secure the tendon to the dual-mode lever arm and the foot and knee were secured. The muscle was then stimulated to contract by placing electrodes adjacent to the sciatic nerve. The optimal length (Lo) was determined based on production of maximum twitch force (Pt), resting muscle length was recorded. The TA was then stimulated to contract (5 – 200Hz, with 2 minutes rest between each contraction) to generate a force frequency curve and maximal tetanic force (Po) was achieved at 150Hz. Absolute force (mN), specific force (mN/mm^2^), and the rate of muscle fatigue and recovery (%) were determined as previously outlined in Garton, *et al*. 2018 [17]. An additional control group of standard chow fed C57/BL6 mice (n = 10) were compared to the *Nf1*_*Prx1*_^*-/-*^ test groups. The operator was blinded to treatment in all cases.

### Tissue collection and histological staining

Mice were euthanized via cervical dislocation and muscles were dissected out and weighed, discarding of overlying fascia and adipose tissue. Muscle tissues were surface coated in Tissue-Tek^®^ O.C.T. Compound (Sakura Finetek USA), placed on a thin piece of tin foil and frozen in liquid nitrogen supercooled isopentane (2-methyl butane) and stored at -80 °C. 8um sections were cut on a Leica CM1950 Clinical Cryostat, and captured on Superfrost™ Plus Microscope Slides (Fisher Scientific, USA) and stored at 4 °C prior to staining.

Oil Red O staining were performed as previously published [6]. Quantification was done using Fiji ImageJ, by quantifying total lipid stained red area as a percentage of total section area.

### Liquid chromatography–mass spectrometry (LC-MS) lipid analysis

Please see the Supporting information (S1 File) for a complete description of lipidomics materials and methods. Lipids for LC-MS analysis were extracted using a modified Bligh Dyer extraction protocol. Lipids were analyzed using Agilent LC 1290 binary pump coupled with Ascentis Express RP amide (50 ×2.1 mm, 1.8u), and separated lipid species were detected using Agilent QQQ 6490 mass spectrometer, using multiple reaction monitoring (MRM) as previously published [6].

For GC-MS based fatty acid analysis, dried samples and dried fatty acid calibration mix were derivatised with 5 μL of Meth-Prep^™^ II (Grace Davison Discovery). The samples were then analyzed on a GC-MS system comprised of a Gerstel 2.5.2 Autosampler, a 7890A Agilent gas chromatograph and a 5975C Agilent quadrupole mass spectrometer (Agilent, Santa Clara, USA) [18, 19].

### Statistical analysis

For Oil Red O analysis, the average lipid area from four sections from n = 10 (study 1) or n = 6 (study 2) individual mice were compared by ANOVA with Tukey's post-hoc multiple comparisons test (multiple groups) or two-tailed Student’s t-test (two groups). For muscle physiology measures, similar parametric comparisons were made using n = 10 mice. Experimental results are expressed as mean ± SEM. P-values of <0.05 were considered statistically significant.

## Results

### Measuring strength, fatigability and muscle recovery in *Nf1*_*Prx1*_^*-/-*^ mice

**Fig 1:**
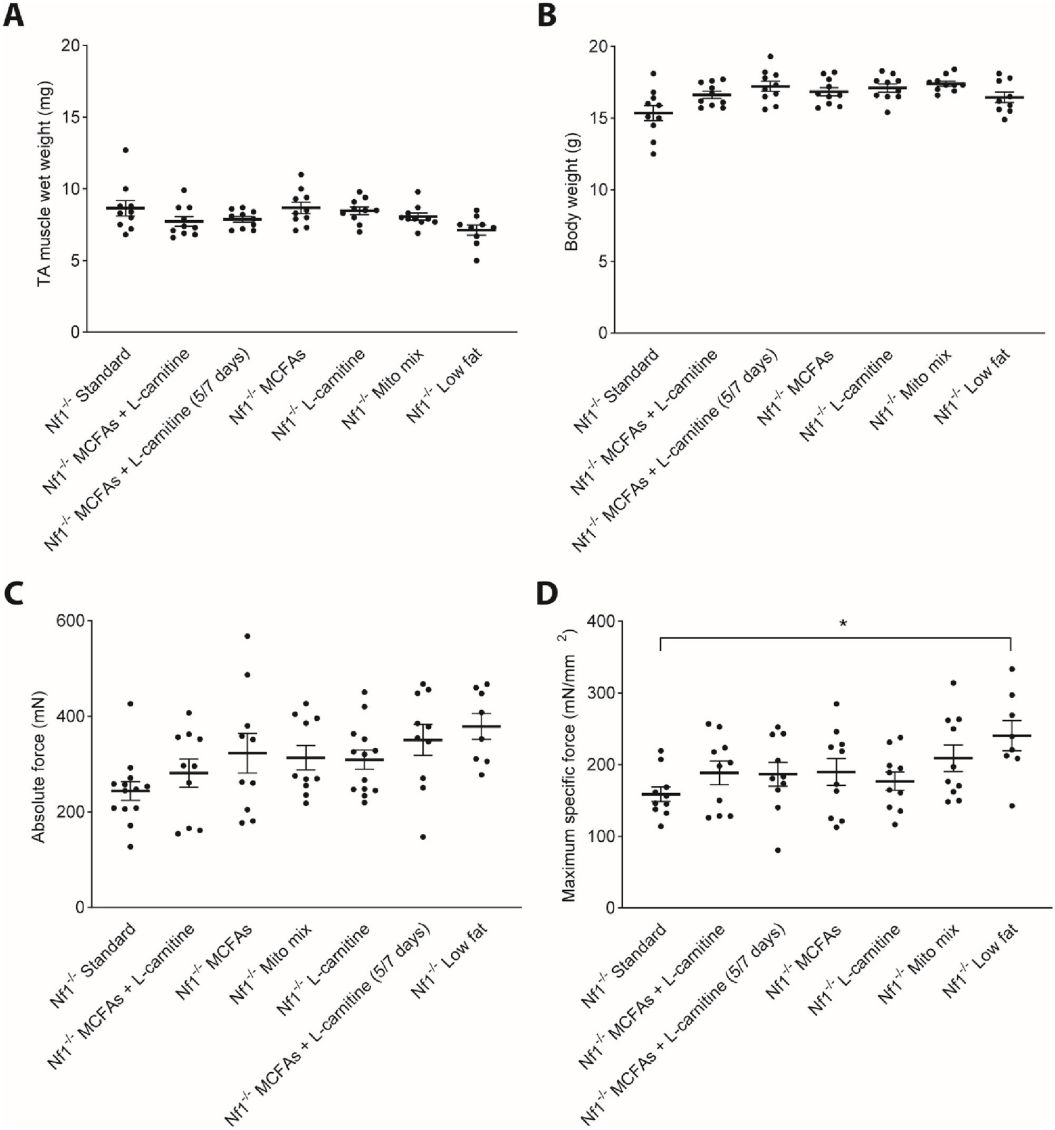
*Nf1Prx1-/-* mice fed a low fat diet have increased maximum specific force without change to TA muscle wet weight. (A) TA muscle wet weight and (B) Body weight remained unchanged following 8 weeks of dietary treatment. (C-D) Low fat feeding resulted in a 66% increase in maximum specific force compared with Nf1_Prx1_^-/-^ fed standard chow. *N* = 10 *in situ* TA muscles per group. Data presented as group mean ± SEM. p-values were assessed by one way ANOVA. *p <0.02.

A prior study using the *Nf1*_*Prx1*_^*-/-*^ mice used grip strength as the primary functional outcome measure [6], however more detailed assessment was sought using *in situ* muscle physiology. Following 8 weeks of *ad libitum* access to the allocated dietary treatments outlined in Table 1, the maximum specific force was measured in all groups of *Nf1*_*Prx1*_^*-/-*^ mice. Notably, the low fat diet regimen yielded a 66% increase in maximal specific force of the TA muscle compared with standard chow (Fig 1D) (*n* = 10 *P* < 0.02). The increased maximum specific force was not associated with changes in TA muscle wet weight or body weight (Fig 1A and 1B). Other diet intervention groups did not demonstrate significant changes compared with standard chow (Fig 1C and 1D).

**Fig 2:**
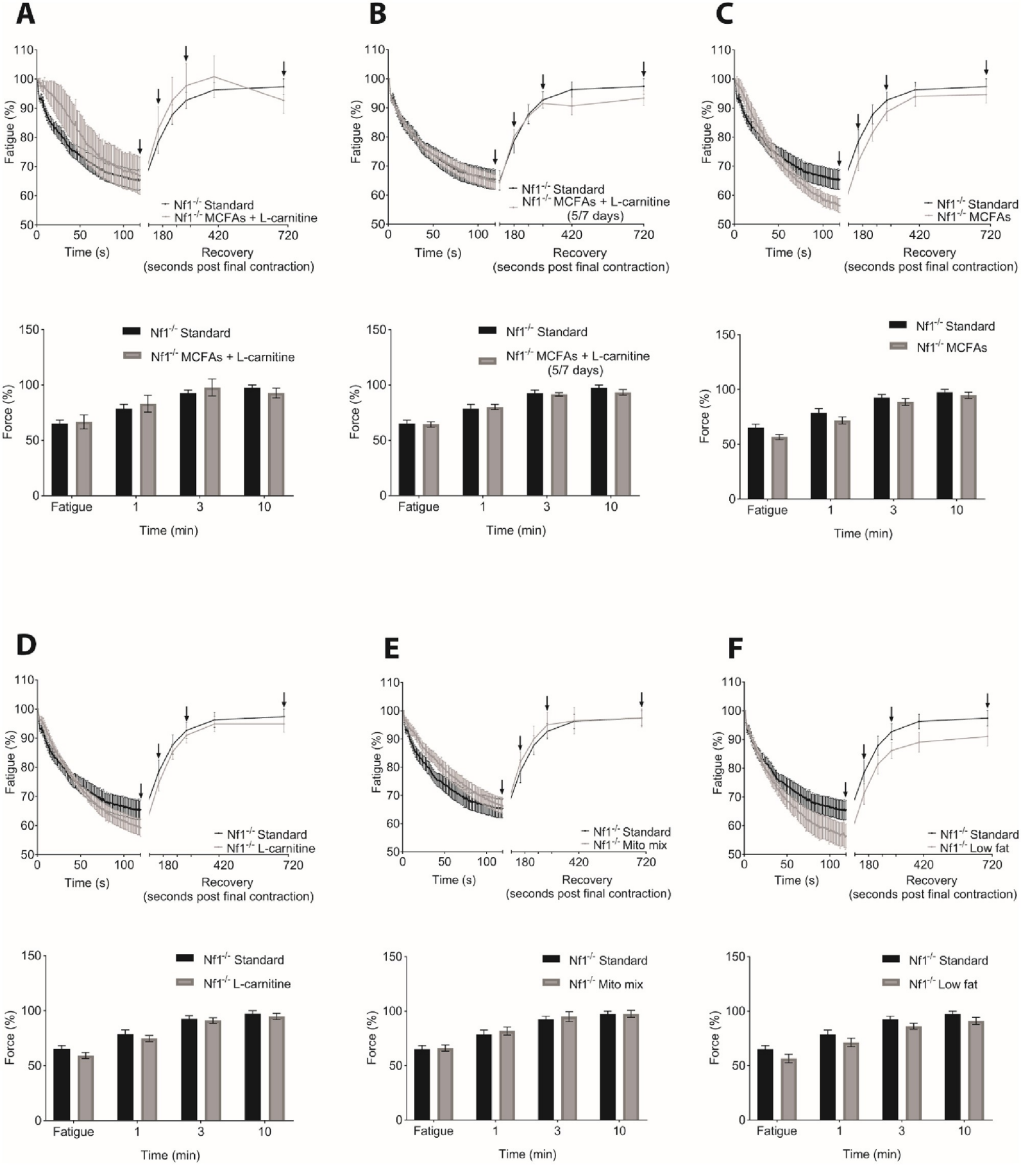
Dietary treatment effects on muscle fatigue and recovery in *Nf1Prx1-/-* TA muscles were unobservable. Fatigue and recovery response of (A) MCFA enriched diet supplemented with L-carnitine (B) MCFA enriched diet supplemented with L-carnitine treatment modelling cheat days (5/7 days) (C) MCFA enriched diet (D) L-carnitine supplementation alone (E) L carnitine in combination with riboflavin, CoQ10 and creatine, a common mitochondrial mix and (F) Low fat diet, at final fatigue measure (120^th^ contraction), 1, 3 and 10 min recovery period (arrows) showed no difference. *N* = 10 *in situ* TA muscles per group. Data presented as group mean ± SEM. p-values were assessed by non-parametric ANOVA.

Analysis of TA muscle fatigability measured following 120 contractions and after 1, 3 and 10 min recovery (Fig 2A–2F) showed an extremely high amount of intragroup variability. No significant differences in muscle function in terms of fatigue and recovery could be detected between groups. Nevertheless, the challenges associated with reliably carrying out the protocol on muscles that were smaller and weaker than WT likely added to the high variance seen in the data.

*Nf1*_*Prx1*_^*-/-*^ mice were characterized relative to wild type mice to confirm reduced wet muscle weight and strength. The wet muscle weight (mg) of the TA in *Nf1*_*Prx1*_^*-/-*^ mice was reduced by 81% compared to WT mice (S1A Fig). Additionally, they exhibited an 83% reduction in absolute force (mN) (S1B Fig).

### Multiple dietary interventions reduced IMCL in *Nf1*_*Prx1*_^*-/-*^ mice

**Fig 3:**
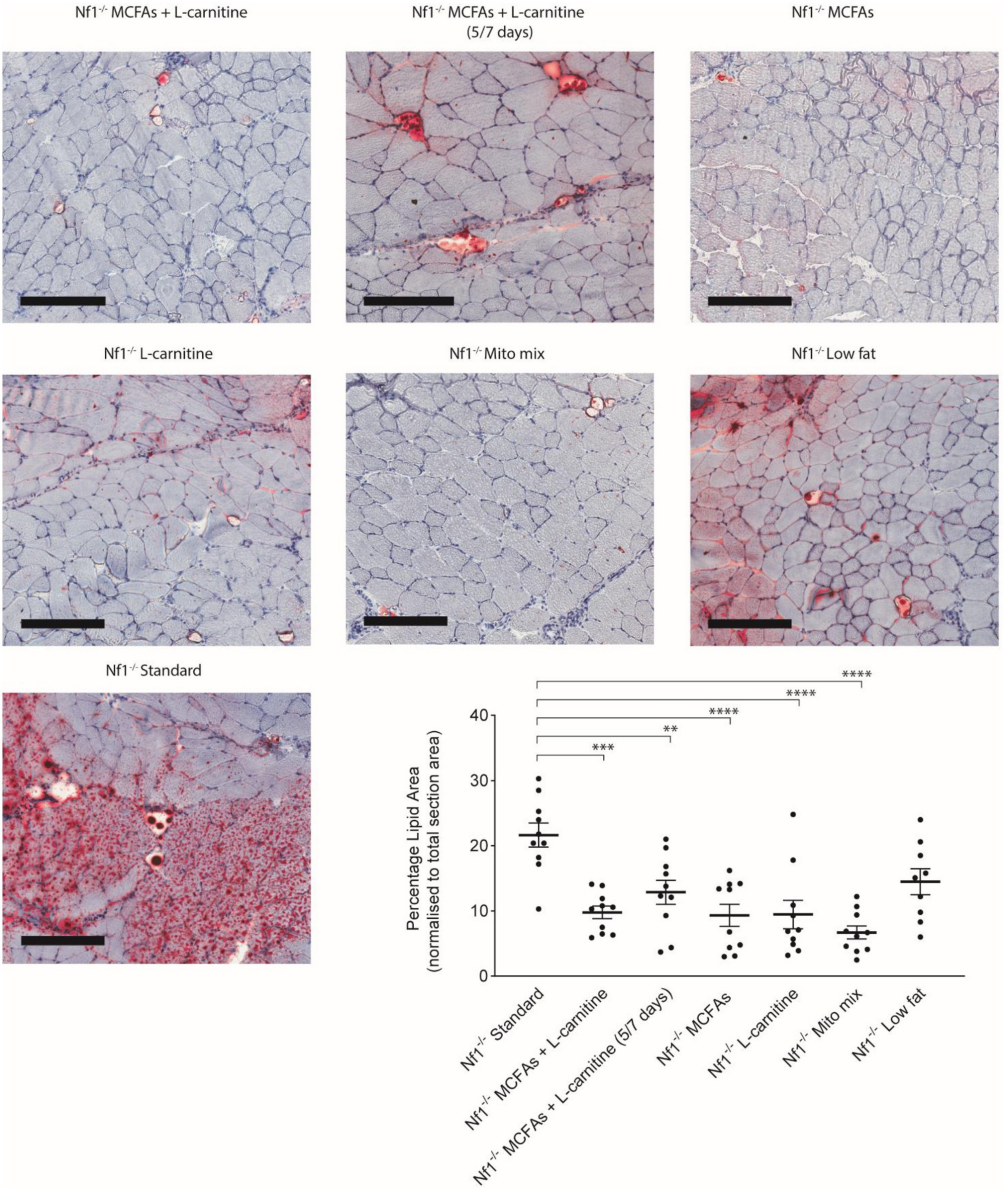
Dietary modifications in *Nf1_Prx1_-/-* mice rescue IMCL accumulation. Histological analysis using Oil Red O showed up to 69% reduction in IMCL accumulation. Scale bar; 200um at 10x magnification, *n* = 10. Data presented as group mean ± SEM. p-values were assessed by one way ANOVA. **p = 0.0083, ***p = 0.0001 and ****p<0.0001.

The major prior finding that prompted this study was that high MCFA diet + L-carnitine could reduce IMCL accumulation in *Nf1*_*Prx1*_^*-/-*^ mice [6]. Analysis of the two components of this treatment separately, as well as modeling cheat days (5/7 days) and a “mito mix” all support the concept of dietary intervention for this condition. Oil Red O staining of sections taken from the mid-belly of the quadriceps was used to show histological changes in lipid droplet density. MCFAs + L-carnitine, MCFAs, L-carnitine and mito mix treatment yielded a 55–69% reduction in IMCL, whereas MCFAs + L-carnitine (5/7 days) gave a 40% reduction (Fig 3) (n = 10, P<0.001). This can be visualized in representative sections (Fig 3). In contrast, the low fat diet did not change IMCL via histology (Fig 3).

### The response to dietary intervention occurs within 4 weeks and subsides after cessation of treatment

**Fig 4:**
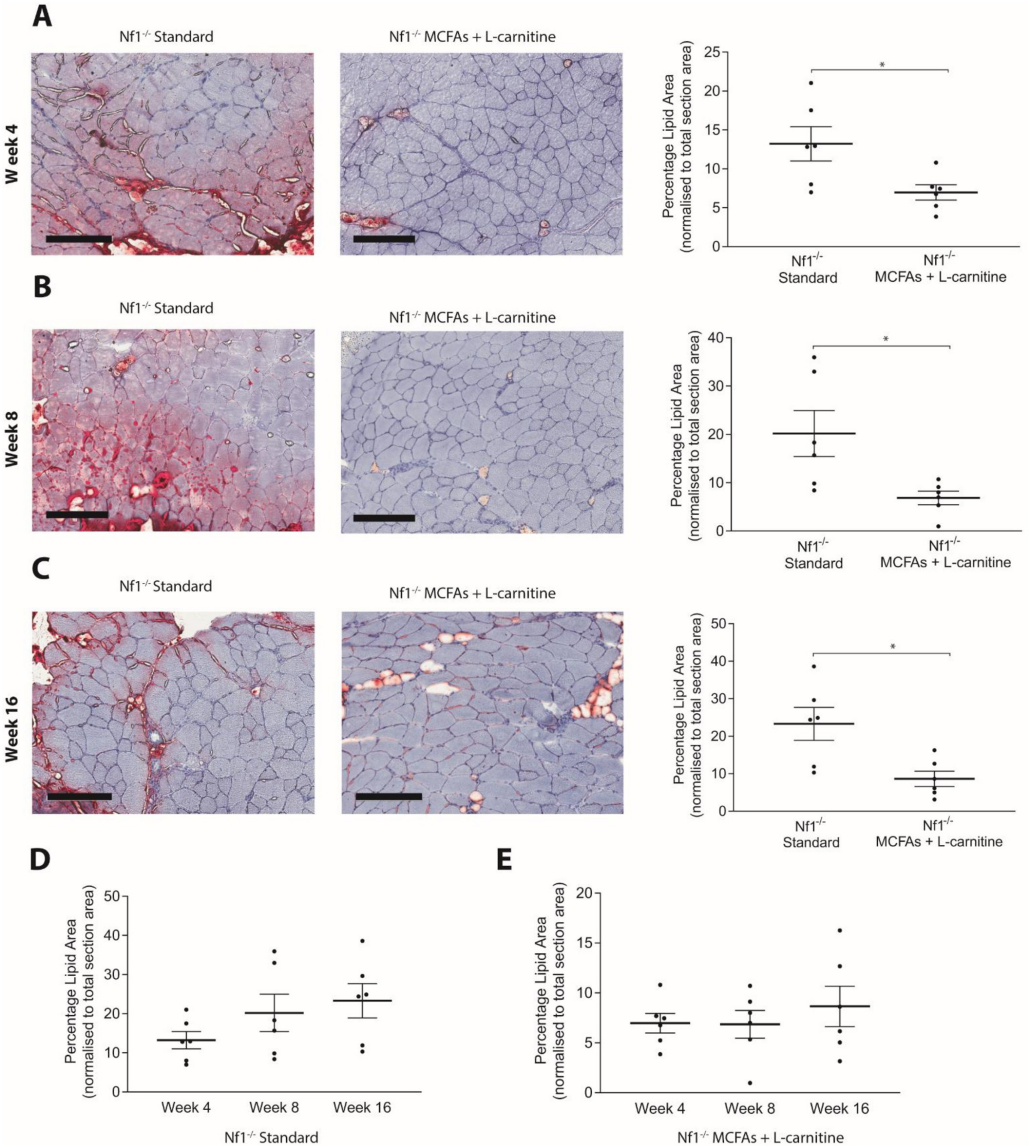
*Nf1_Prx1_-/-* mice fed MCFA + L-carnitine chow show reduced IMCL accumulation within 4 weeks of treatment. A significant reduction of IMCL accumulation was observed in quadriceps muscle of *Nf1*_*Prx1*_^*-/-*^ mice fed MCFA + L-carnitine chow compared to standard chow fed *Nf1*_*Prx1*_^*-/-*^ mice at all time points; (A) 4 weeks (B) 8 weeks and (C) 16 weeks of treatment. (D) IMCL accumulation did not significantly increase over 12 weeks in standard chow fed *Nf1*_*Prx1*_^*-/-*^ mice. (E) IMCL reduction plateaued after 4 weeks of MCFA + L-carnitine treatment in *Nf1*_*Prx1*_^*-/-*^ mice. Scale bar; 200um at 10x magnification, *n* = 6. Data presented as group mean ± SEM. p-values were assessed by one way ANOVA.

**Fig 5:**
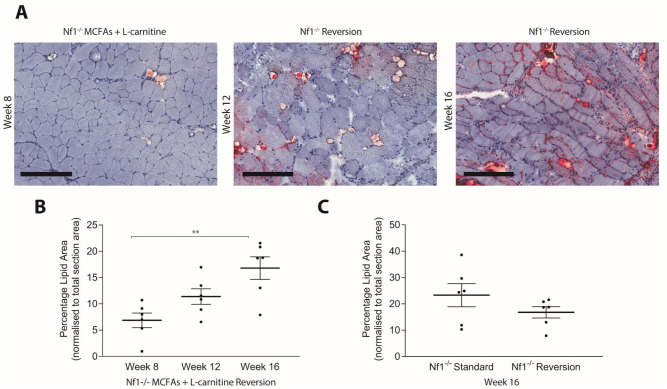
IMCL re-builds following reversion from MCFA + L-carnitine chow to standard chow. (A) Representative histopathology of quadriceps muscle from *Nf1*_*Prx1*_^*-/-*^ mice following reversion to standard chow. (B) Cessation of MCFA + L-carnitine chow causes IMCL to return over 8 week period. (C) IMCL levels return to levels of standard chow fed *Nf1*_*Prx1*_^*-/-*^ mice following 8 week of dietary reversion. Scale bar; 200um at 10x magnification, *n* = 6. Data presented as group mean ± SEM. p-values were assessed by one way ANOVA.

Longitudinal assessment of *Nf1*_*Prx1*_^*-/-*^ mice fed a high MCFA diet + L-carnitine diet was performed. Histological staining confirmed a reduction of IMCL as early as 4 weeks of treatment (n = 6, p<0.03) (Fig 4A). This reduction was consistent with prior findings with dietary intervention at a single time point of 8 weeks [6]. Moreover, the reduction persisted concomitant with treatment out to the final time point of 16 weeks (n = 6, p<0.02) (Fig 4B and 4C). For mice that received MCFAs + L-carnitine for 8 weeks and were then reverted to standard chow, IMCL was found to re-accumulate after a further 4 and 8 weeks (n = 6, p<0.003) (Fig 5). These data suggest that dietary changes need to be maintained in order to prevent the build-up of new IMCL.

### Changes in lipidome profile in response to *Nf1* deficiency and dietary intervention

To help elucidate the mechanism underlying the changes in IMCL accumulation, muscle samples underwent lipid mass spectrometry analysis for a variety of lipid species including triglycerides (TGs), diglycerides (DGs) and free fatty acids. Three test groups: WT mice on standard chow, *Nf1*_*Prx1*_^*-/-*^ mice on standard chow, and *Nf1*_*Prx1*_^*-/-*^ mice on modified chow (MCFAs + L-carnitine) were examined after 8 weeks of dietary intervention. This utilized homogenized quadriceps muscle with all associated subcutaneous adipose tissue carefully removed.

**Fig 6:**
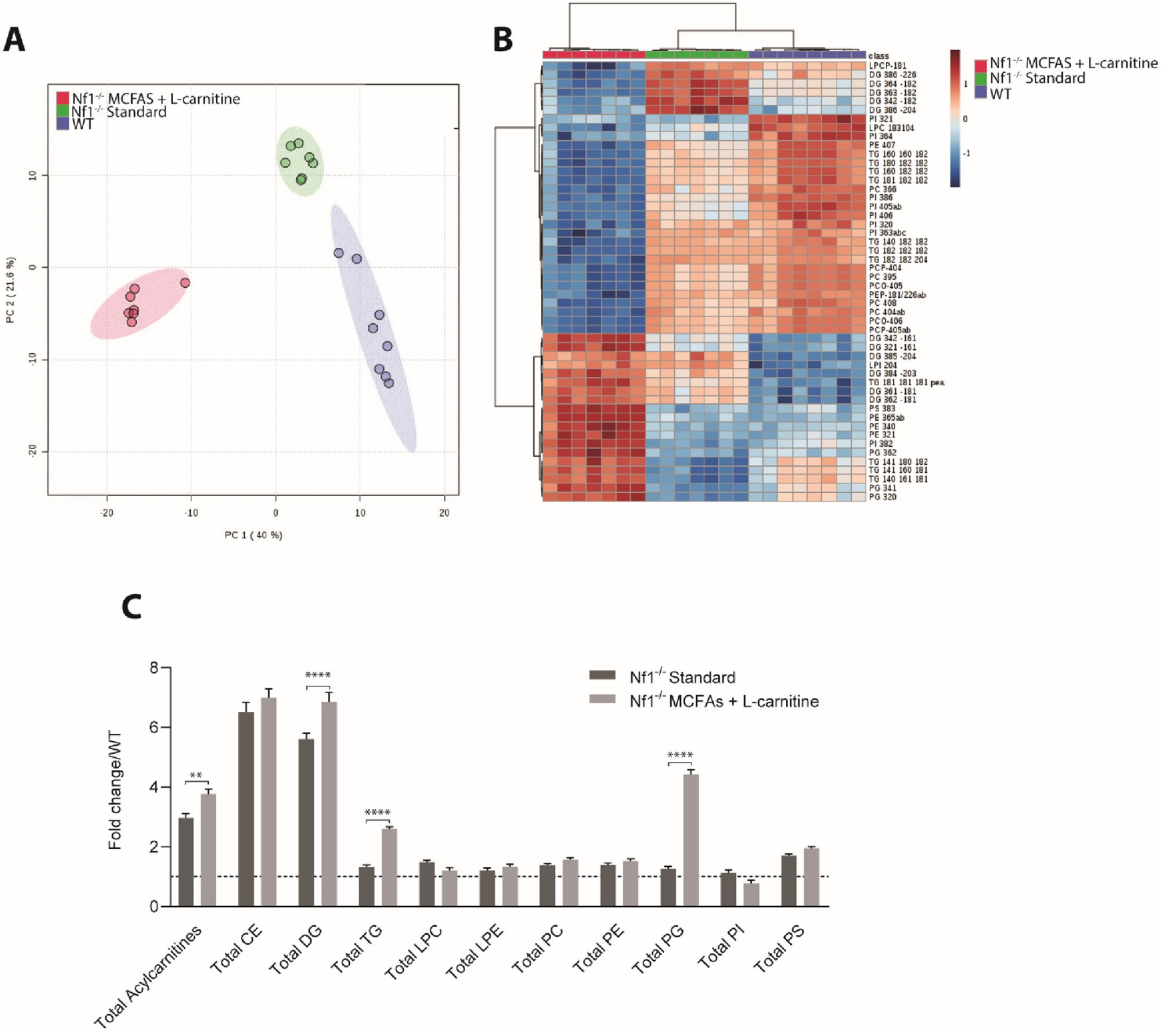
Lipidomics analysis of whole quadriceps of MCFA + L-carnitine fed *Nf1_Prx1_-/-* mice confirms dietary intervention can alter lipid metabolism. (A) Principal-component analysis of LC-MS data showed group clustering and separation of lipid data between WT, *Nf1*_*Prx1*_^*-/-*^ mice fed standard chow and *Nf1*_*Prx1*_^*-/-*^ fed MCFA + L-carnitine chow. The first two components captured >60% of the variance across the dataset (PC1: 40%, PC2: 21.6%). (B) Heat map analysis of the top 50 lipid species selected based on fold change suggests increased TG and DG species. (C) Total DG, TG and PG are increased in whole quadriceps of *Nf1*_*Prx1*_^*-/-*^ fed MCFA + L-carnitine chow. Total acylcarnitines are elevated in *Nf1*_*Prx1*_^*-/-*^ mice, which were exacerbated upon MCFA dietary enrichment and carnitine supplementation. *n* = 8 quadriceps muscle samples analyzed for all lipidomics studies. Data presented as group mean ± SEM fold change compared to WT. p-values were assessed by ANOVA. **** p <0.0001 ** p<0.003. CE; Cholesterol esters, DG; Diglycerides, TG; triglycerides, LPC; Lysophosphatidylcholine, LPE; Lysophosphatidylethanolamine, PC; Phosphatidylcholine, PE; Phosphatidylethanolamine, PG; Phosphatidylglycerol, PI; Phosphatidylinositol, PS; Phosphatidylserine.

Principal Component Analysis (PCA) was used to determine whether the samples clustered into distinct groups, which indeed was the case (Fig 6A). The first two components captured >60% of the variance across the dataset (PC1: 40%, PC2: 21.6%). This supports the concept that not only genotype but also diet leads to consistent and separable changes in muscle lipidome profile. Hierarchical clustering analysis and heat map visualization of the top 50 lipid species selected based on fold change suggested substantive increases in species of DG, including DG 16:0, DG 16:1, DG 18:1, species of TG, including TG16:1, TG 18:1, and species of phosphatidylglycerol (PG), including PG 34:1, PG 34:2 and PG 36:2 in modified chow treated *Nf1*_*Prx1*_^*-/-*^ mice (Fig 6B). Total TG, total DG and total PGs were all significantly elevated in *Nf1*_*Prx1*_^*-/-*^ mice fed the modified chow (n = 8, P <0.0001) (Fig 6C). In contrast, total CE, LPC, LPE, PC, PE, PI and PS were unaltered (Fig 6C). Fatty acid analysis using GC-MS confirmed increases in several FA species including palmitoleic acid (C16:1), oleic Acid (C18:1n9c) and eicosapentaenoic acid (C20:5n3) in modified chow-fed *Nf1*_*Prx1*_^*-/-*^ mice (Table 3).

**Table 3:** GC-MS analysis reveals significant increase of fatty acids in whole quadriceps of modified diet fed Nf1_Prx1_-/- mice. *n* = 8 quadriceps muscle samples per group were analyzed. Data presented as group mean ± SEM fold change compared to WT. p-values were assessed by ANOVA.

GC-MS				
Fatty Acid Analysis				
Fatty Acid	Fold increase in Nf1^-/-^ Standard muscle	*P* value	Fold increase in Nf1^-/-^ MCFAs + L-carnitine	*P* value
Palmitoleic Acid (C16:1)	5.12	<0.001	20.51	0.001
Oleic Acid (C18:1n9c)	5.91	<0.001	14.35	0.006
cis-5,8,11,14,17-Eicosapentaenoic acid (C20:5n3)	1.86	0.002	14.12	<0.001
Myristoleic Acid (C14:1)	2.15	0.003	7.91	0.002
Myristic Acid (C14:0)	3.16	0.001	7.07	0.001
Palmitic Acid (C16:0)	3.54	<0.001	5.95	0.017

**Fig 7:**
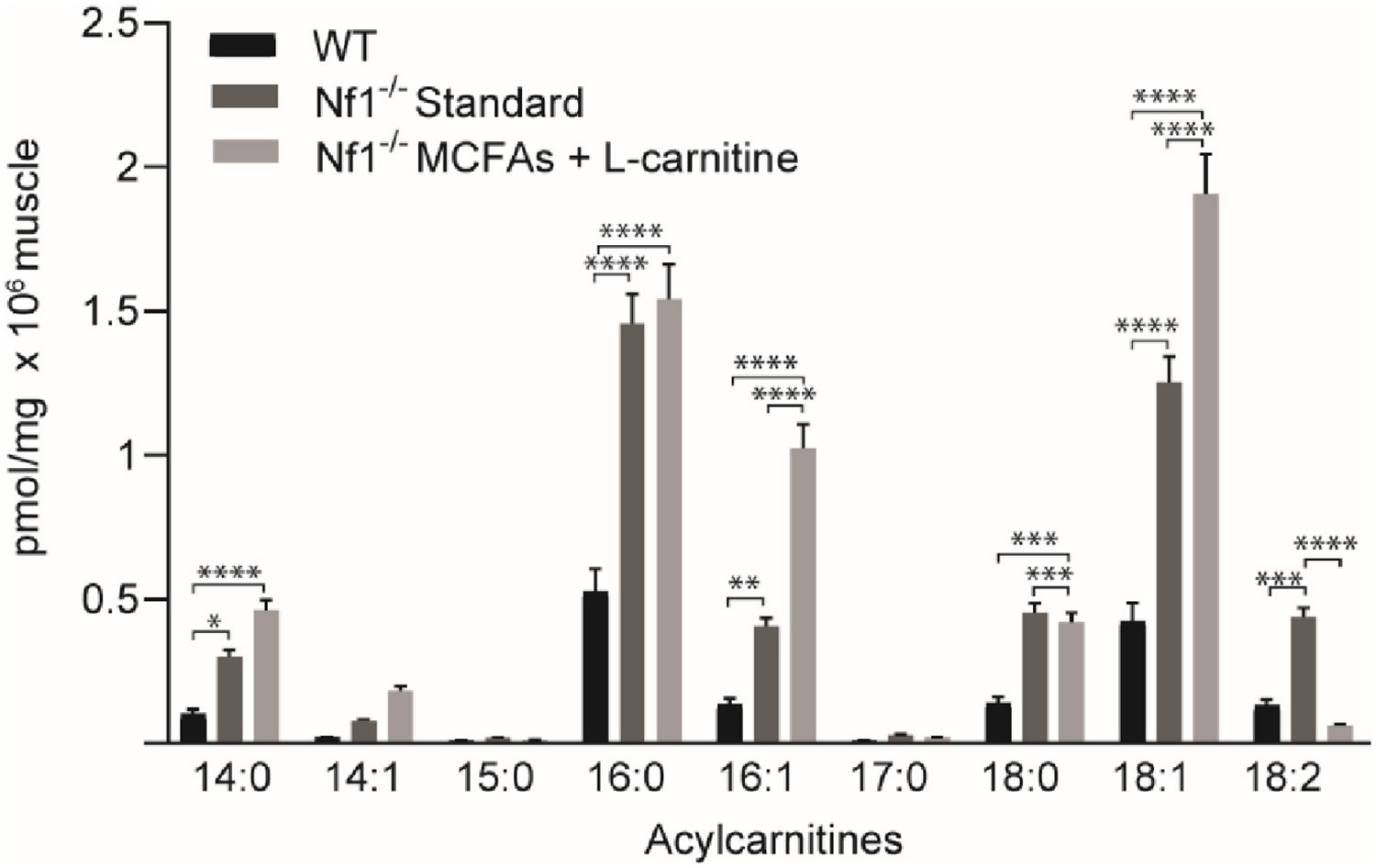
Acylcarnitines are elevated in *Nf1_Prx1_-/-* mice compared to WT. C14:0, C16:0, C16:1, C18:0, C18:1 and C18:2 are significantly increased in whole quadriceps muscle samples of *Nf1*_*Prx1*_^*-/-*^ mice compared to WT, which are further exacerbated (excluding C18:2) in modified diet fed *Nf1*_*Prx1*_^*-/-*^ mice. *n* = 8 quadriceps muscle samples per group were analyzed. Data presented as group mean ± SEM. p-values were assessed by one way ANOVA. **** p<0.0001 ** p<0.003. C14:0 and C14:1; Tetradecanoylcarnitines, C16:0 and C16:1; hexadecenoylcarnitines, C18:0 and C18:1; octadecenoylcarnitines and C18:2; octadecadienoylcarnitine.

Strikingly, total acylcarnitines were elevated in *Nf1*_*Prx1*_^*-/-*^ mice compared to WT mice (n = 8, p<0.003) (Fig 7). Detailed analysis of individual acylcarnitine species revealed significantly increased levels of acylcarnitine 14:0 (n = 8, p<0.024), 16:0 (n = 8, p<0.0001), 16:1 (n = 8, p<0.0013), 18:0 (n = 8, p<), 18.1 (n = 8, p<0.0001) and 18:2 (n = 8, p<0.0002). Acylcarnitine 16:1 and 18.1 (n = 8, p<0.0001) were further increased with modified chow feeding (Fig 7).

The full lipidomics data set is included in the Supporting information (S2 File).

## Discussion

Murine studies have previously shown that *Nf1* deficiency is associated with accumulation of IMCL high in LCFAs. This study was prompted by our prior finding that a modified diet enriched with MCFAs and supplemented with L-carnitine could decrease muscle IMCL and improve grip strength in *Nf1*_*Prx1*_^*-/-*^ mice [5, 6]. This study confirms and builds upon these findings.

Our first major finding was the relative increase in strength on a low fat diet. This was an unexpected finding, and notably not aligned to any changes in muscle lipid. A low fat diet is commonly recommended to LSM patients, and it has been demonstrated to decrease liver size and normalize hepatic enzymes [20]. Liver dysfunction and physical function has not been adequately studied, however there is growing evidence to suggest that liver dysfunction causes impaired muscle protein synthesis and decline of muscle strength [21], which could be rectified by a low fat diet [21]. A second major finding was establishing that L-carnitine alone and increased MCFAs alone could both produce reductions in IMCL. This has translational applications for clinical trials and therapy as L-carnitine can already be readily purchased in capsule form as a dietary supplement, and is simpler to medicate than a complex dietary program. Lastly, the reappearance of IMCL following halting of the modified diet suggests that any clinical therapy will need to be ongoing to maintain benefits in individuals with NF1.

Moving forward, L-carnitine supplementation represents a promising therapeutic intervention for individuals with NF1 and concerns about muscle strength and fatigue. L-carnitine is a well-established treatment for LSM’s, including primary carnitine deficiency [11]. These patients can show a rapid reversal of clinical symptoms within one month [22–24] and, consistent with our murine data, L-carnitine needs to be maintained to provide ongoing symptomatic relief [9, 25]. While it can be challenging to extrapolate clinical timelines from animal data, the effects in our model were seen rapidly–as early as 4 weeks in the mice.

Nevertheless, the mechanism of L-carnitine on lipid metabolism and the lipidome has not been extensively studied, particularly in *NF1*-deficient muscle. The capacity of L-carnitine to reduce IMCL in *Nf1*_*Prx1*_^*-/-*^ mice suggests a redirection from storage pathways to lipolysis. This is supported by an increased fatty acid content in muscle of mice fed the modified diet, as shown by GC-MS data. In cultured adipocytes, the addition of L-carnitine supplementation has been shown to stimulate lipolysis by the induction of lipolytic gene expression, including hormone sensitive lipase, carnitine palmitoyltransferase Ia (CPT-1a) and Acyl-CoA oxidase, and suppression of adipogenic genes, including PPARγ [26]. In a zebrafish model, L-carnitine supplementation similarly resulted in significantly increased CPT1 and decreased fatty acid synthase (FAS) expression [27]. The latter is particularly notable as *Nf1*_*Prx1*_^*-/-*^ muscle exhibits increased FAS expression and decreased CPT1 levels [5].

While the lipidomics analysis revealed a range of changes within the *Nf1*_*Prx1*_^*-/-*^ genotype, of particular note was the increase in acylcarnitines (particularly C16 and C18:1). Substantially elevated acylcarnitines have been previously associated with disorders of mitochondrial fatty acid oxidation and organic academia’s [28, 29]. Furthermore, elevated plasma C16 and C18:1 acylcarnitines are the formal diagnostic criteria for carnitine-acylcarnitine translocase deficiency [30], and support diagnosis of carnitine palmitoyltransferase II deficiency [31]. The acylcarnitine levels were further increased with dietary modification. Thus one possibility is that aberrant NF1-Ras signaling may lead to downstream changes in CPT1 activity as the primary enzyme that exchanges the CoA moiety from long-chain acyl-CoAs for carnitine to generate acylcarnitines [32].

While the *Nf1*_*Prx1*_^*-/-*^ mouse model has proven useful for biochemical analysis, it remains challenging as a model to study muscle function. The mouse features partial-to-complete fusion of the hip joint [4, 33], which impairs locomotion and causes secondary reductions in loading and strength over time. The *Nf1*_*Prx1*_^*-/-*^ hind limb muscles were proportionally smaller than the forelimb muscle when compared to WT mice (S2 Fig). Prior grip strength tests primarily utilize the forelimb muscles. In contrast, the *in situ* strength and fatigue testing focused on the hind limb TA muscle, which presents the greatest reduction in muscle size (-83%) compared to WT TA. Additionally, the variability of hip fusion affected our ability position their hind limbs, and subsequently altered the dorsal location of the mouse for *in situ* muscle physiology analysis. The resultant variability in the *in situ* muscle physiology data was challenging to draw definitive conclusions from. Indeed a post-hoc power analysis suggested n ≥ 54 mice are required to achieve 80% power for a genotype/diet effect, which is not feasible due to severity of the model and poor breeding of the strain.

In conclusion, data from this preclinical study supports the concept that NF1 features a metabolic dysregulation that can be ameliorated by dietary intervention. In particular, L-carnitine supplementation appears to be a feasible and promising option for human trials as it is inexpensive and well-tolerated. Such clinical studies would likely be superior options to examine functional effects on muscle in a longitudinal manner than the challenging *Nf1*_*Prx1*_^*-/-*^ mouse model. The lipidome analysis suggests that Nf1 produces profound changes in the lipidome that can be altered by dietary intervention suggestive of an increase in muscle lipolysis. Moreover, the murine data suggests some evidence for dysregulated carnitine metabolism in Nf1, however this will need to be further addressed using clinical samples. While this study has focused specifically on muscle, these data also raise the possibility that the Nf1 regulation of metabolism may affect other tissues, e.g. bone [34] and could have broader implications for the treatment of the condition.

## Supporting information

S1 Fig *Nf1Prx1-/-* mice demonstrated significant deficits in muscle size and force production compared to WT. (A) *Nf1*_*Prx1*_^*-/-*^ TA muscle wet weight was reduced by 81% compared to WT and (B) *Nf1*_*Prx1*_^*-/-*^ TA force was reduced by 83% compared to WT. *N* = 10 *in situ* TA muscles per group. Data presented as group mean ± SEM.
(TIF)

S2 Fig Cartilaginous fusion of the hip joints is a characteristic feature of the *Nf1Prx1-/-* mouse model that varies between complete and partial fusion. (A) Lower extremity representative X-ray images of *Nf1*_*Prx1*_^*-/-*^ mice to demonstrate leg position variability in dorsal placement due to variability in hip fusion. (B) Hind limb muscle wet weight is reduced by 61–83% in the *Nf1*_*Prx1*_^*-/-*^ mouse model compared to WT.
(TIF)

S1 File Complete description of lipidomics materials and methods. (PDF)

S2 File Full lipidomics data set. (XLSX)
