# Correction: Evaluating modified diets and dietary supplement therapies for reducing muscle lipid accumulation and improving muscle function in neurofibromatosis type 1 (NF1)

**DOI:** 10.1371/journal.pone.0249380

**Published:** 2021-03-24

**Authors:** The PLOS ONE Staff

## Notice of republication

This article was republished on October 5, 2020, to include a Publisher’s Note. The Publisher’s Note was added to clarify that the article is a pre-registered research article and to provide information about the assessment process administered by Children’s Tumor Foundation (CTF) and *PLOS ONE*. Please download the article again to view the correct version. The originally published, uncorrected article and the republished, corrected article are provided here for reference.

## Supporting information

S1 File Originally published, uncorrected article. (PDF)

S2 File Republished corrected article. (PDF)
