# Supplementary figures and images for: Evaluating modified diets and dietary supplement therapies for reducing muscle lipid accumulation and improving muscle function in neurofibromatosis type 1 (NF1)
Source: PLoS One. 2020 Aug 10;15(8):e0237097. doi: 10.1371/journal.pone.0237097 (PMC7446925; doi:10.1371/journal.pone.0237097)

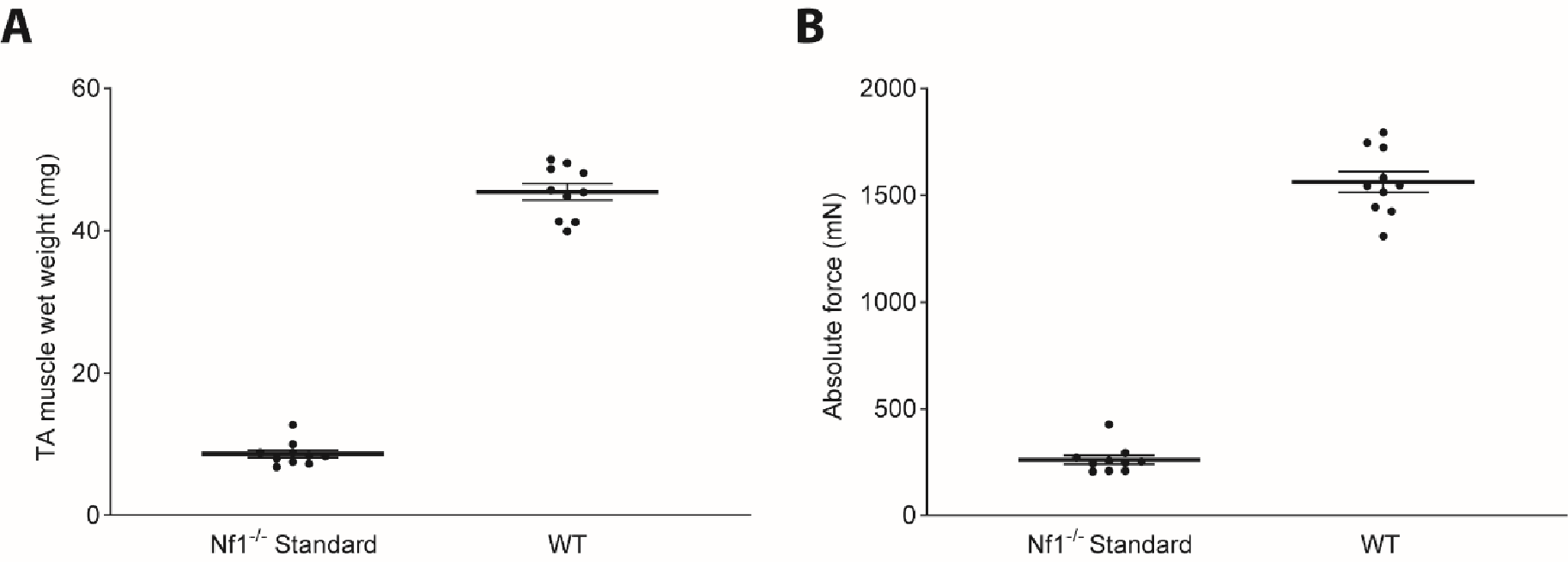

Supplement: S1 Fig — (A) Nf1Prx1-/- TA muscle wet weight was reduced by 81% compared to WT and (B) Nf1Prx1-/- TA force was reduced by 83% compared to WT. N = 10 in situ TA muscles per group. Data presented as group mean ± SEM. (TIF) [file pone.0237097.s001.tif]

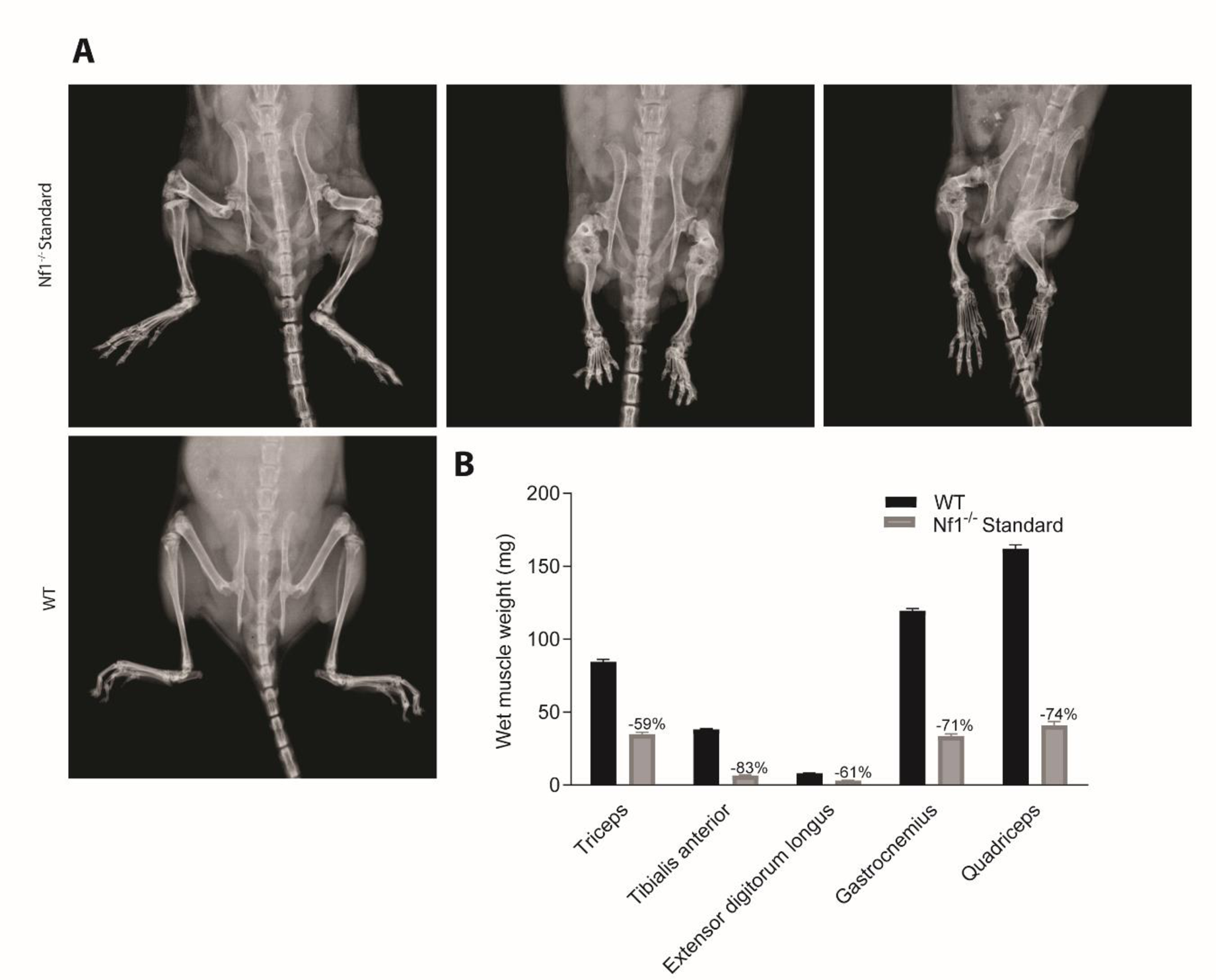

Supplement: S2 Fig — (A) Lower extremity representative X-ray images of Nf1Prx1-/- mice to demonstrate leg position variability in dorsal placement due to variability in hip fusion. (B) Hind limb muscle wet weight is reduced by 61–83% in the Nf1Prx1-/- mouse model compared to WT. (TIF) [file pone.0237097.s002.tif]
