# Supplementary material for: Evaluating modified diets and dietary supplement therapies for reducing muscle lipid accumulation and improving muscle function in neurofibromatosis type 1 (NF1)
Source: PLoS One. 2020 Aug 10;15(8):e0237097. doi: 10.1371/journal.pone.0237097 (PMC7446925; doi:10.1371/journal.pone.0237097)
Supplement: S1 File — (PDF) [file pone.0237097.s003.pdf]

Metabolomic Data Analysis with MetaboAnalyst 4.0

Name: guest8335145726651203987

February 19, 2020

1 Data Processing and Normalization

1.1 Reading and Processing the Raw Data

MetaboAnalyst accepts a variety of data types generated in metabolomic studies, including compound concentration data, binned NMR/MS spectra data, NMR/MS peak list data, as well as MS spectra (NetCDF, mzXML, mzDATA). Users need to specify the data types when uploading their data in order for MetaboAnalyst to select the correct algorithm to process them. Table 1 summarizes the result of the data processing steps.

1.1.1 Reading Peak Intensity Table

The peak intensity table should be uploaded in comma separated values (.csv) format. Samples can be in rows or columns, with class labels immediately following the sample IDs.

Samples are in rows and features in columns The uploaded file is in comma separated values (.csv) format. The uploaded data file contains 29 (samples) by 369 (peaks(mz/rt)) data matrix.

1.1.2 Data Integrity Check

Before data analysis, a data integrity check is performed to make sure that all the necessary information has been collected. The class labels must be present and contain only two classes. If samples are paired, the class label must be from -n/2 to -1 for one group, and 1 to n/2 for the other group (n is the sample number and must be an even number). Class labels with same absolute value are assumed to be pairs. Compound concentration or peak intensity values should all be non-negative numbers. By default, all missing values, zeros and negative values will be replaced by the half of the minimum positive value found within the data (see next section)

1.1.3 Missing value imputations

Too many zeroes or missing values will cause difficulties for downstream analysis. MetaboAnalyst offers several different methods for this purpose. The default method replaces all the missing and zero values with a small values (the half of the minimum positive values in the original data) assuming to be the detection limit. The assumption of this approach is that most missing values are caused by low abundance metabolites (i.e.below the detection limit). In addition, since zero values may cause problem for data normalization (i.e. log), they are also replaced with this small value. User can also specify other methods, such as replace by mean/median, or use K-Nearest Neighbours (KNN), Probabilistic PCA (PPCA), Bayesian PCA (BPCA) method, Singular Value Decomposition (SVD) method to impute the missing values [1]. Please choose the one that is the most appropriate for your data.

[1] Stacklies W, Redestig H, Scholz M, Walther D, Selbig J. *pcaMethods: a bioconductor package, providing PCA methods for incomplete data.*, Bioinformatics 2007 23(9):1164-1167

Zero or missing variables were replaced with a small value: 0.05

1.1.4 Data Filtering

The purpose of the data filtering is to identify and remove variables that are unlikely to be of use when modeling the data. No phenotype information are used in the filtering process, so the result can be used with any downstream analysis. This step can usually improves the results. Data filter is strongly recommended for datasets with large number of variables (> 250) datasets contain much noise (i.e.chemometrics data). Filtering can usually improve your results[2].

For data with number of variables < 250, this step will reduce 5% of variables; For variable number between 250 and 500, 10% of variables will be removed; For variable number bwteen 500 and 1000, 25% of variables will be removed; And 40% of variabled will be removed for data with over 1000 variables. The None option is only for less than 5000 features. Over that, if you choose None, the IQR filter will still be applied. In addition, the maximum allowed number of variables is ***10000***

No non-QC based data filtering was applied

Table 1: Summary of data processing results

	Features (positive)	Missing/Zero	Features (processed)
1790_KOMOD	357	12	360
1809_KOMOD	358	11	360
1812_KOMOD	358	11	360
1813_KOMOD	358	11	360
1819_KOMOD	359	10	360
1820_KOMOD	357	12	360
1822_KOMOD	357	12	360
1776_KOCON	357	12	360
1786_KOCON	358	11	360
1787_KOCON	359	10	360
1792_KOCON	359	10	360
1799_KOCON	357	12	360
1803_KOCON	357	12	360
1805_KOCON	358	11	360
1805_KOCONR	357	12	360
PBQC6	355	14	360
PBQC7	354	15	360
PBQCeq_1	353	16	360
PBQCeq_2	358	11	360
PBQCeq_3	352	17	360
PBQCeq_4	355	14	360
2427_WT	352	17	360
2428_WT	359	10	360
2429_WT	356	13	360
2430_WT	358	11	360
2431_WT	357	12	360
2437_WT	358	11	360
2438_WT	356	13	360
2439_WT	358	11	360

[2]Hackstadt AJ, Hess AM.*Filtering for increased power for microarray data analysis*, BMC Bioinformatics. 2009; 10: 11.

1.2 Data Normalization

The data is stored as a table with one sample per row and one variable (bin/peak/metabolite) per column. The normalization procedures implemented below are grouped into four categories. Sample specific normalization allows users to manually adjust concentrations based on biological inputs (i.e. volume, mass); row-wise normalization allows general-purpose adjustment for differences among samples; data transformation and scaling are two different approaches to make features more comparable. You can use one or combine both to achieve better results.

The normalization consists of the following options:

1. Row-wise procedures:
   - Sample specific normalization (i.e. normalize by dry weight, volume)
   - Normalization by the sum
   - Normalization by the sample median
   - Normalization by a reference sample (probabilistic quotient normalization)[3]
   - Normalization by a pooled or average sample from a particular group
   - Normalization by a reference feature (i.e. creatinine, internal control)
   - Quantile normalization
2. Data transformation :
   - Generalized log transformation (glog 2)
   - Cube root transformation
3. Data scaling:
   - Mean centering (mean-centered only)
   - Auto scaling (mean-centered and divided by standard deviation of each variable)
   - Pareto scaling (mean-centered and divided by the square root of standard deviation of each variable)
   - Range scaling (mean-centered and divided by the value range of each variable)

Figure 1 shows the effects before and after normalization.

[3]Dieterle F, Ross A, Schlotterbeck G, Senn H. *Probabilistic quotient normalization as robust method to account for dilution of complex biological mixtures. Application in 1H NMR metabonomics*, 2006, Anal Chem 78 (13);4281 - 4290

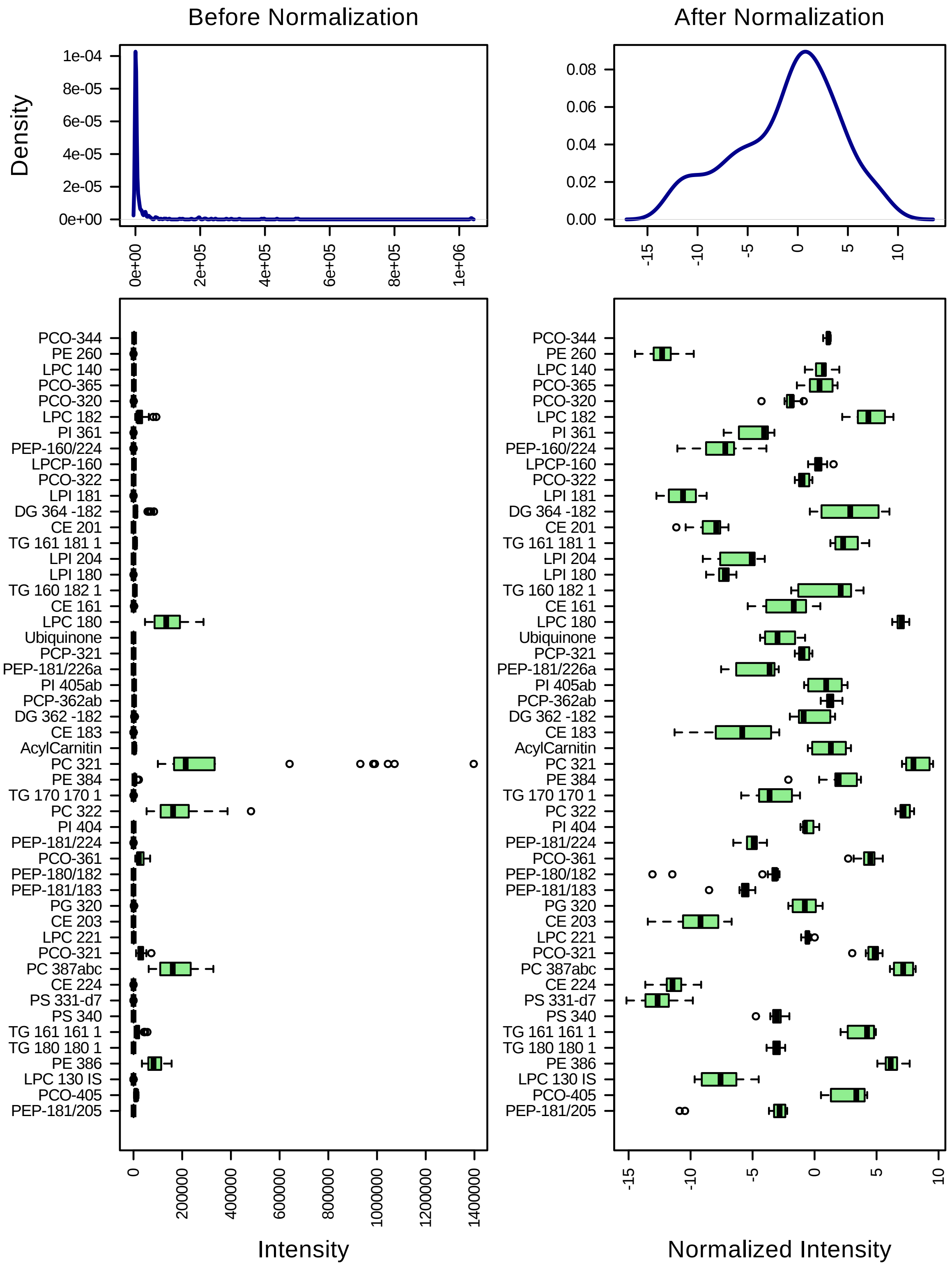

Figure 1: Box plots and kernel density plots before and after normalization. The boxplots show at most 50 features due to space limit. The density plots are based on all samples. Selected methods : Row-wise normalization: Normalization to sample median; Data transformation: Log Normalization; Data scaling: N/A.

2 Statistical and Machine Learning Data Analysis

MetaboAnalyst offers a variety of methods commonly used in metabolomic data analyses. They include:

1. Univariate analysis methods:
   - Fold Change Analysis
   - T-tests
   - Volcano Plot
   - One-way ANOVA and post-hoc analysis
   - Correlation analysis
2. Multivariate analysis methods:
   - Principal Component Analysis (PCA)
   - Partial Least Squares - Discriminant Analysis (PLS-DA)
3. Robust Feature Selection Methods in microarray studies
   - Significance Analysis of Microarray (SAM)
   - Empirical Bayesian Analysis of Microarray (EBAM)
4. Clustering Analysis
   - Hierarchical Clustering
     - Dendrogram
     - Heatmap
   - Partitional Clustering
     - K-means Clustering
     - Self-Organizing Map (SOM)
5. Supervised Classification and Feature Selection methods
   - Random Forest
   - Support Vector Machine (SVM)

`Please note:  some advanced methods are available only for two-group sample analyais.`

2.1 One-way ANOVA

Univariate analysis methods are the most common methods used for exploratory data analysis. For multi-group analysis, MetaboAnalyst provides one-way Analysis of Variance (ANOVA). As ANOVA only tells whether the overall comparison is significant or not, it is usually followed by post-hoc analyses in order to identify which two levels are different. MetaboAnalyst provides two most commonly used methods for this purpose - Fisher's least significant difference method (Fisher's LSD) and Tukey's Honestly Significant Difference (Tukey's HSD). The univariate analyses provide a preliminary overview about features that are potentially significant in discriminating the conditions under study.

Figure 2 shows the important features identified by ANOVA analysis. Table 2 shows the details of these features. The `post-hoc Sig. Comparison` column shows the comparisons between different levels that are significant given the p value threshold.

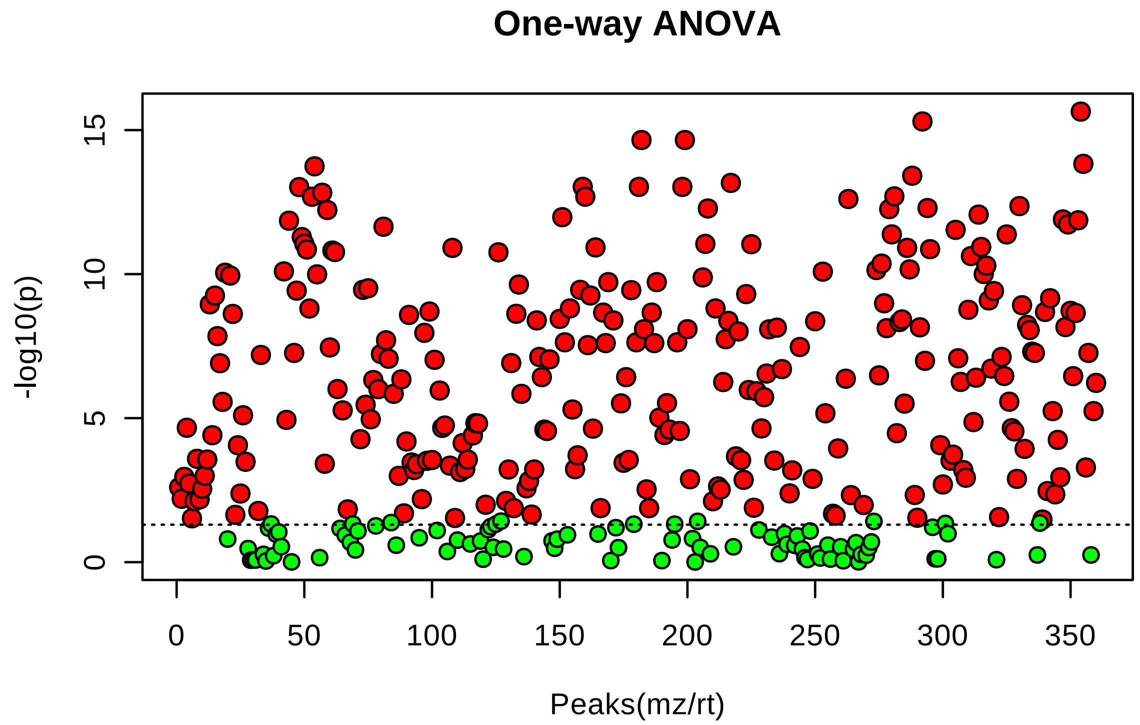

Figure 2: Important features selected by ANOVA plot with p value threshold 0.05.

Table 2: Top 50 features identified by One-way ANOVA and post-hoc analysis

	Peaks(mz/rt)	f.value	p.value	-log10(p)	FDR	Fisher's LSD
1	TG 182 182 182	411.42	2.2827e-16	15.642	8.2178e-14	Nf1-/- (standard) - Nf1-/- (mod diet); WT - Nf1-/- (mod diet)
2	PI 386	377.93	5.0177e-16	15.299	9.0318e-14	Nf1-/- (standard) - Nf1-/- (mod diet); WT - Nf1-/- (mod diet); W
3	PCO-406	321.93	2.2114e-15	14.655	1.9902e-13	Nf1-/- (standard) - Nf1-/- (mod diet); WT - Nf1-/- (mod diet); W
4	PCP-405ab	321.93	2.2114e-15	14.655	1.9902e-13	Nf1-/- (standard) - Nf1-/- (mod diet); WT - Nf1-/- (mod diet); W
5	TG 182 182 204	261.69	1.4871e-14	13.828	1.0707e-12	Nf1-/- (standard) - Nf1-/- (mod diet); WT - Nf1-/- (mod diet)
6	DG 364 -182	256.14	1.8094e-14	13.742	1.0856e-12	Nf1-/- (standard) - Nf1-/- (mod diet); WT - Nf1-/- (mod diet); N
7	PI 382	235.87	3.8459e-14	13.415	1.9779e-12	Nf1-/- (mod diet) - Nf1-/- (standard); Nf1-/- (mod diet) - WT; W
8	PE 365ab	221.52	6.8194e-14	13.166	2.8541e-12	Nf1-/- (mod diet) - Nf1-/- (standard); Nf1-/- (mod diet) - WT
9	PC 395	213.92	9.3682e-14	13.028	2.8541e-12	Nf1-/- (standard) - Nf1-/- (mod diet); WT - Nf1-/- (mod diet); W
10	PCO-405	213.92	9.3682e-14	13.028	2.8541e-12	Nf1-/- (standard) - Nf1-/- (mod diet); WT - Nf1-/- (mod diet); W
11	PCP-404	213.92	9.3682e-14	13.028	2.8541e-12	Nf1-/- (standard) - Nf1-/- (mod diet); WT - Nf1-/- (mod diet); W
12	DG 342 -161	213.56	9.5136e-14	13.022	2.8541e-12	Nf1-/- (mod diet) - Nf1-/- (standard); Nf1-/- (mod diet) - WT; N
13	DG 384 -203	203.15	1.4978e-13	12.825	4.1478e-12	Nf1-/- (mod diet) - Nf1-/- (standard); Nf1-/- (mod diet) - WT; N
14	PI 321	196.81	1.9971e-13	12.700	4.6442e-12	WT - Nf1-/- (mod diet); WT - Nf1-/- (standard)
15	PC 404ab	196.17	2.0575e-13	12.687	4.6442e-12	Nf1-/- (standard) - Nf1-/- (mod diet); WT - Nf1-/- (mod diet); W
16	DG 363 -182	196.10	2.0641e-13	12.685	4.6442e-12	Nf1-/- (standard) - Nf1-/- (mod diet); WT - Nf1-/- (mod diet); N
17	PEP-181/226ab	192.29	2.4648e-13	12.608	5.2197e-12	Nf1-/- (standard) - Nf1-/- (mod diet); WT - Nf1-/- (mod diet)
18	TG 160 182 182	180.61	4.3441e-13	12.362	8.6882e-12	Nf1-/- (standard) - Nf1-/- (mod diet); WT - Nf1-/- (mod diet); W
19	PI 405ab	177.47	5.0870e-13	12.294	9.4759e-12	Nf1-/- (standard) - Nf1-/- (mod diet); WT - Nf1-/- (mod diet); W
20	PE 340	176.80	5.2644e-13	12.279	9.4759e-12	Nf1-/- (mod diet) - Nf1-/- (standard); Nf1-/- (mod diet) - WT
21	PG 362	175.78	5.5468e-13	12.256	9.5088e-12	Nf1-/- (mod diet) - Nf1-/- (standard); Nf1-/- (mod diet) - WT; W
22	DG 385 -204	174.39	5.9577e-13	12.225	9.7490e-12	Nf1-/- (mod diet) - WT; Nf1-/- (standard) - WT
23	TG 140 182 182	167.48	8.5716e-13	12.067	1.3416e-11	Nf1-/- (standard) - Nf1-/- (mod diet); WT - Nf1-/- (mod diet)
24	PC 366	163.66	1.0544e-12	11.977	1.5816e-11	Nf1-/- (standard) - Nf1-/- (mod diet); WT - Nf1-/- (mod diet); W
25	TG 180 182 182	160.39	1.2641e-12	11.898	1.8203e-11	Nf1-/- (standard) - Nf1-/- (mod diet); WT - Nf1-/- (mod diet); W
26	TG 181 182 182	159.23	1.3490e-12	11.870	1.8678e-11	Nf1-/- (standard) - Nf1-/- (mod diet); WT - Nf1-/- (mod diet); W
27	DG 321 -161	158.56	1.4013e-12	11.853	1.8684e-11	Nf1-/- (mod diet) - Nf1-/- (standard); Nf1-/- (mod diet) - WT; N
28	TG 181 181 181 peak 2	153.38	1.8863e-12	11.724	2.4252e-11	Nf1-/- (mod diet) - Nf1-/- (standard); Nf1-/- (mod diet) - WT; N
29	LPC 183104	150.30	2.2615e-12	11.646	2.8074e-11	Nf1-/- (standard) - Nf1-/- (mod diet); WT - Nf1-/- (mod diet); W
30	PS 383	146.12	2.9098e-12	11.536	3.4917e-11	Nf1-/- (mod diet) - Nf1-/- (standard); Nf1-/- (mod diet) - WT
31	PI 320	140.31	4.1745e-12	11.379	4.7170e-11	Nf1-/- (standard) - Nf1-/- (mod diet); WT - Nf1-/- (mod diet); W
32	TG 160 160 182	140.25	4.1929e-12	11.377	4.7170e-11	Nf1-/- (standard) - Nf1-/- (mod diet); WT - Nf1-/- (mod diet); W
33	DG 342 -182	136.96	5.1769e-12	11.286	5.6476e-11	Nf1-/- (standard) - Nf1-/- (mod diet); WT - Nf1-/- (mod diet); N
34	DG 361 -181	129.00	8.7999e-12	11.056	9.1611e-11	Nf1-/- (mod diet) - Nf1-/- (standard); Nf1-/- (mod diet) - WT; N
35	PE 321	128.79	8.9291e-12	11.049	9.1611e-11	Nf1-/- (mod diet) - Nf1-/- (standard); Nf1-/- (mod diet) - WT
36	PE 407	128.42	9.1611e-12	11.038	9.1611e-11	Nf1-/- (standard) - Nf1-/- (mod diet); WT - Nf1-/- (mod diet); W
37	TG 141 160 181	125.29	1.1391e-11	10.943	1.1075e-10	Nf1-/- (mod diet) - Nf1-/- (standard); Nf1-/- (mod diet) - WT; W
38	PC 408	124.73	1.1849e-11	10.926	1.1075e-10	Nf1-/- (standard) - Nf1-/- (mod diet); WT - Nf1-/- (mod diet); W
39	PI 363abc	124.22	1.2286e-11	10.911	1.1075e-10	Nf1-/- (standard) - Nf1-/- (mod diet); WT - Nf1-/- (mod diet)
40	LPCP-181	124.20	1.2305e-11	10.910	1.1075e-10	Nf1-/- (standard) - Nf1-/- (mod diet); WT - Nf1-/- (mod diet); N
41	PI 406	122.79	1.3607e-11	10.866	1.1947e-10	Nf1-/- (standard) - Nf1-/- (mod diet); WT - Nf1-/- (mod diet); W
42	DG 362 -181	122.41	1.3985e-11	10.854	1.1988e-10	Nf1-/- (mod diet) - Nf1-/- (standard); Nf1-/- (mod diet) - WT; N
43	DG 386 -204	121.26	1.5201e-11	10.818	1.2726e-10	Nf1-/- (standard) - Nf1-/- (mod diet); WT - Nf1-/- (mod diet); N
44	DG 386 -226	119.62	1.7135e-11	10.766	1.4008e-10	Nf1-/- (standard) - Nf1-/- (mod diet); WT - Nf1-/- (mod diet); N
45	LPI 204	119.33	1.7510e-11	10.757	1.4008e-10	Nf1-/- (mod diet) - WT; Nf1-/- (standard) - WT
46	TG 140 161 181	115.34	2.3610e-11	10.627	1.8478e-10	Nf1-/- (mod diet) - Nf1-/- (standard); Nf1-/- (mod diet) - WT; W
47	PG 341	107.68	4.3081e-11	10.366	3.2998e-10	Nf1-/- (mod diet) - Nf1-/- (standard); Nf1-/- (mod diet) - WT; W
48	TG 141 180 182	105.69	5.0707e-11	10.295	3.8031e-10	Nf1-/- (mod diet) - Nf1-/- (standard); Nf1-/- (mod diet) - WT; W
49	PI 364	102.09	6.8522e-11	10.164	5.0342e-10	Nf1-/- (standard) - Nf1-/- (mod diet); WT - Nf1-/- (mod diet); W
50	PG 320	101.48	7.2222e-11	10.141	5.2000e-10	Nf1-/- (mod diet) - Nf1-/- (standard); Nf1-/- (mod diet) - WT; W

2.2 Principal Component Analysis (PCA)

PCA is an unsupervised method aiming to find the directions that best explain the variance in a data set (X) without referring to class labels (Y). The data are summarized into much fewer variables called *scores* which are weighted average of the original variables. The weighting profiles are called *loadings*. The PCA analysis is performed using the `prcomp` package. The calculation is based on singular value decomposition.

The Rscript `chemometrics.R` is required. Figure 3 is pairwise score plots providing an overview of the various seperation patterns among the most significant PCs; Figure 4 is the scree plot showing the variances explained by the selected PCs; Figure 5 shows the 2-D scores plot between selected PCs; Figure 6 shows the 3-D scores plot between selected PCs; Figure 7 shows the loadings plot between the selected PCs; Figure 8 shows the biplot between the selected PCs.

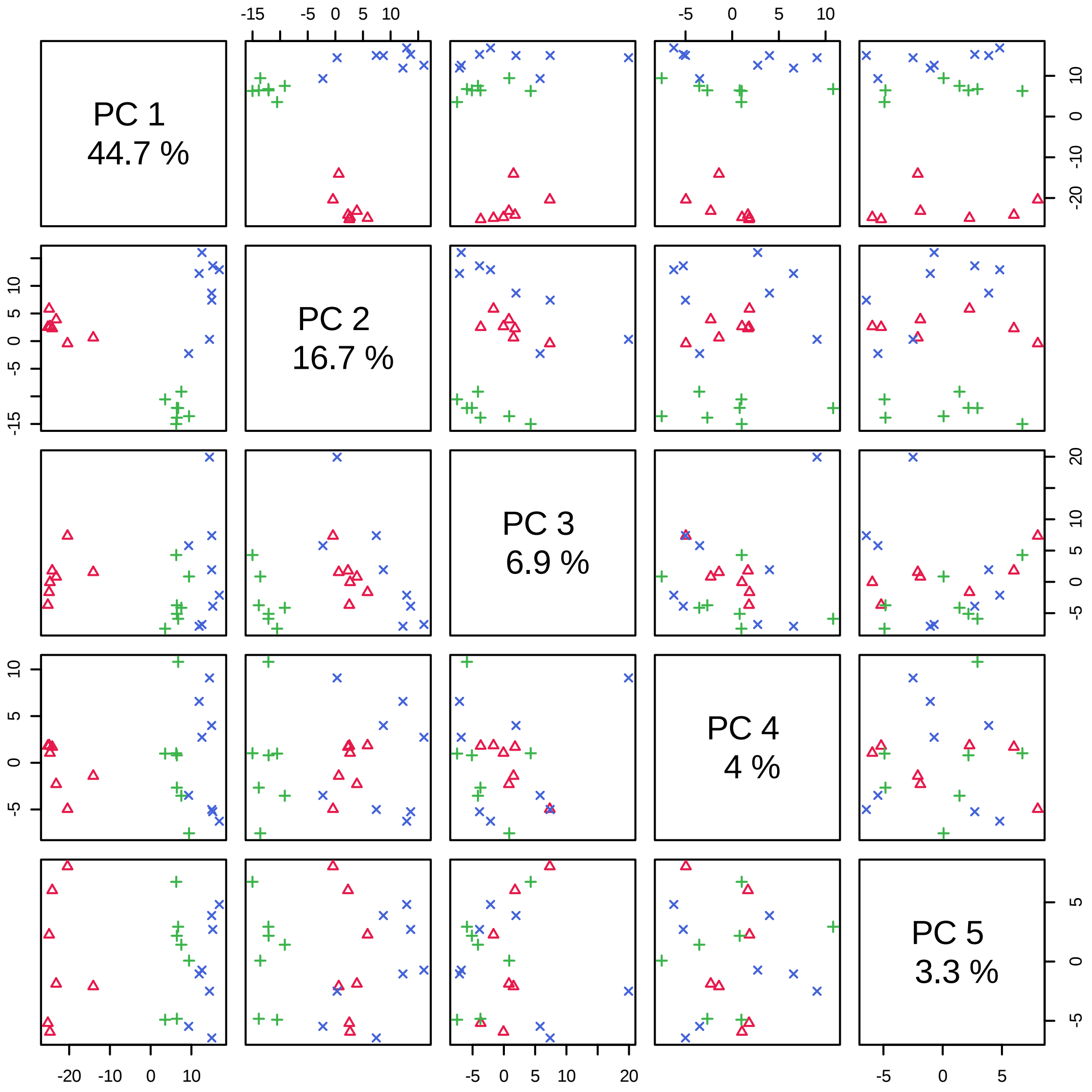

Figure 3: Pairwise score plots between the selected PCs. The explained variance of each PC is shown in the corresponding diagonal cell.

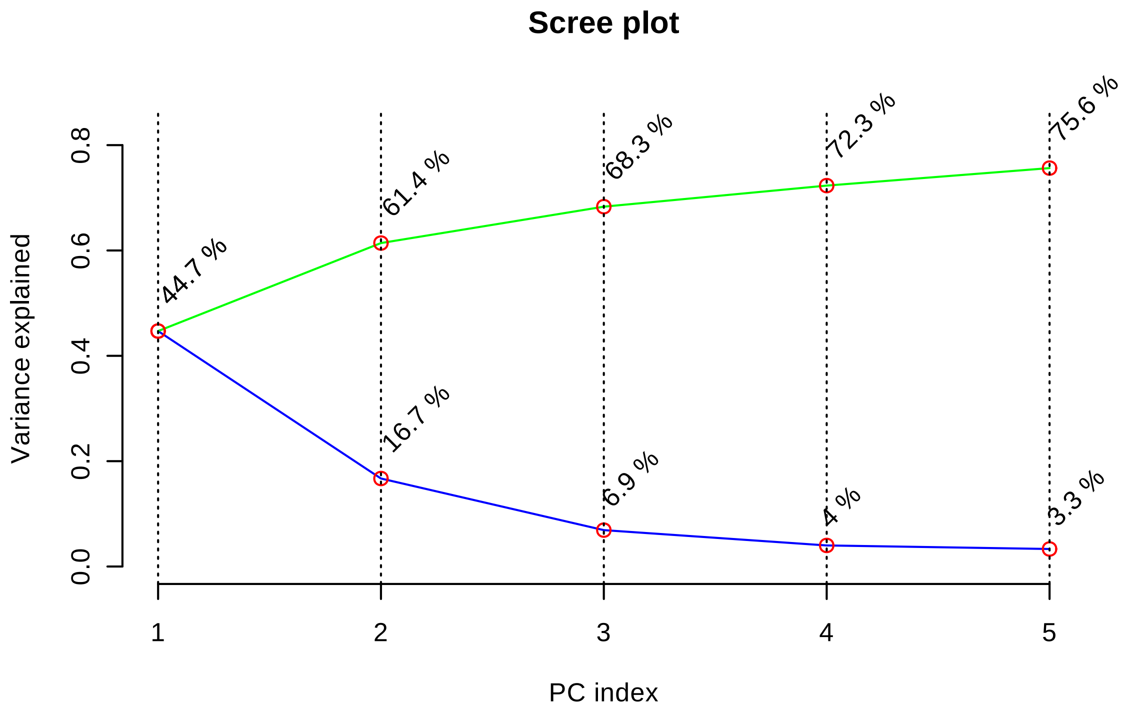

Figure 4: Scree plot shows the variance explained by PCs. The green line on top shows the accumulated variance explained; the blue line underneath shows the variance explained by individual PC.

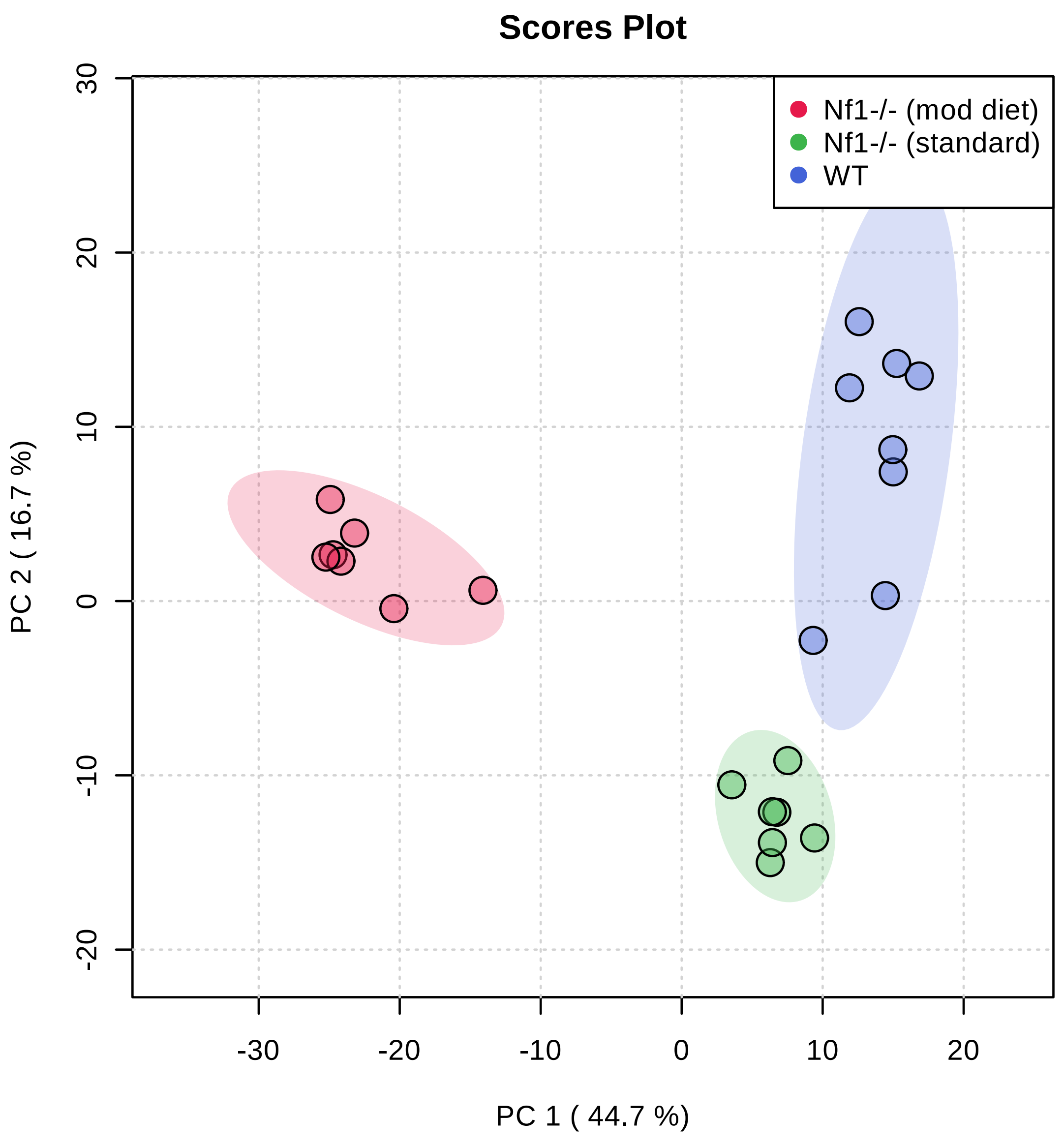

Figure 5: Scores plot between the selected PCs. The explained variances are shown in brackets.

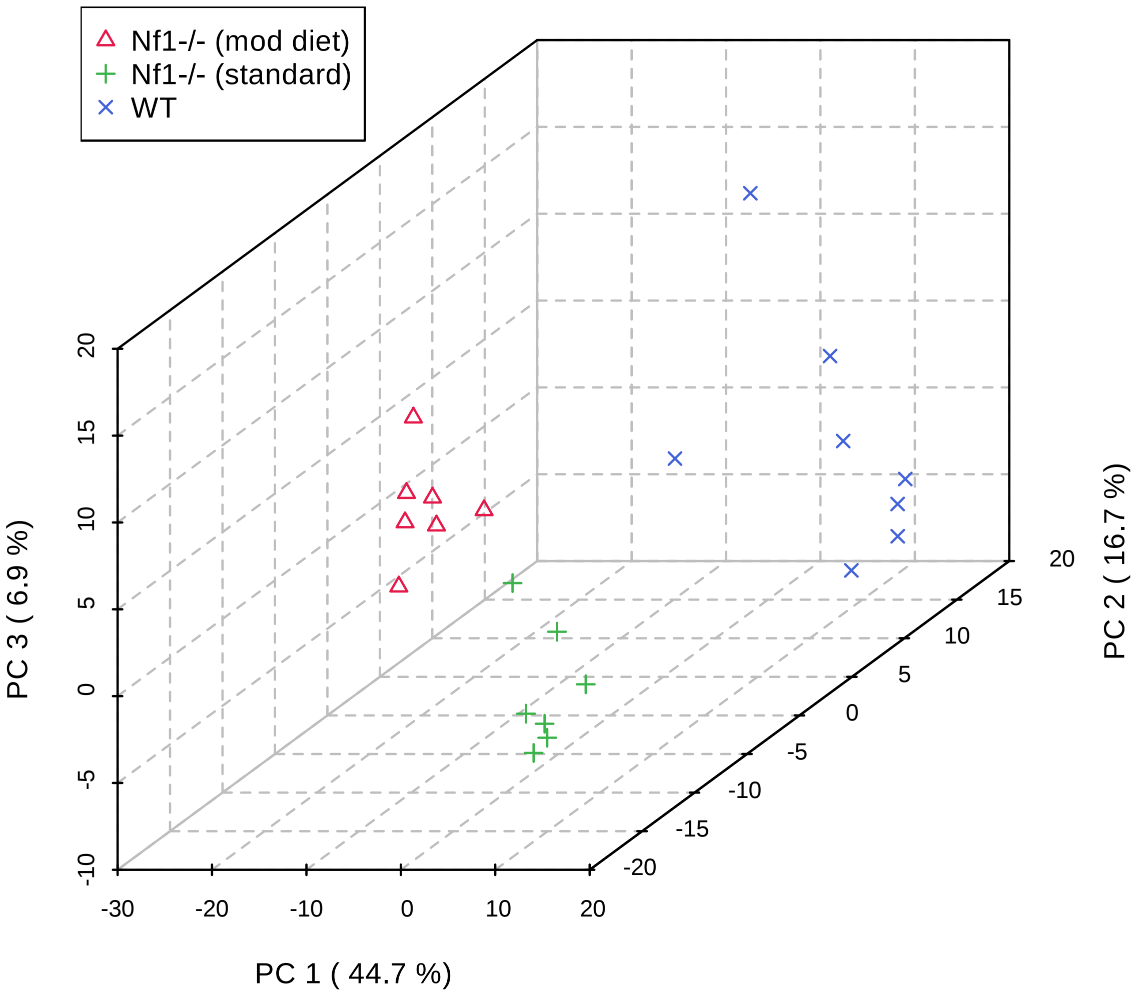

Figure 6: 3D score plot between the selected PCs. The explained variances are shown in brackets.

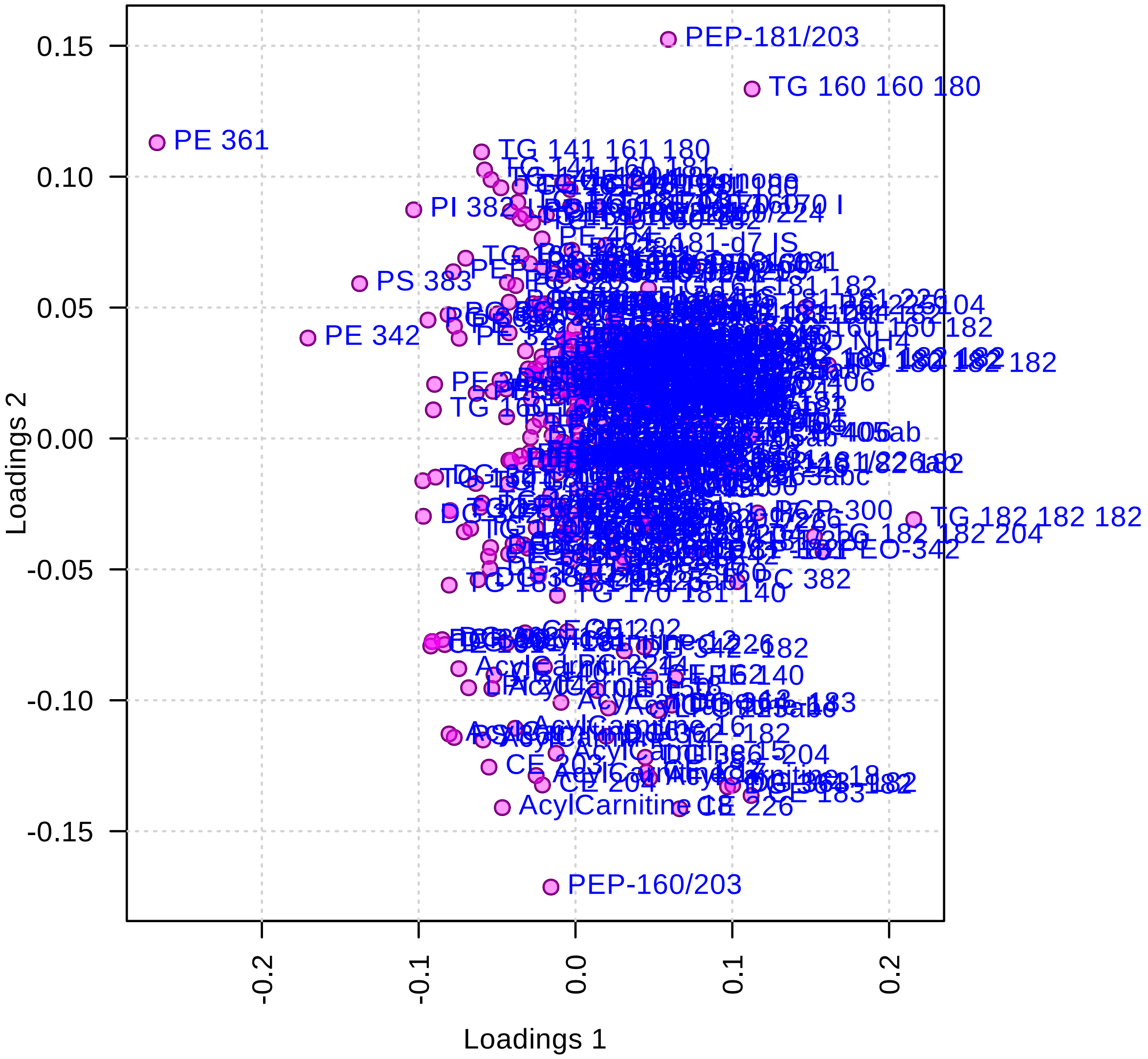

Figure 7: Loadings plot for the selected PCs.

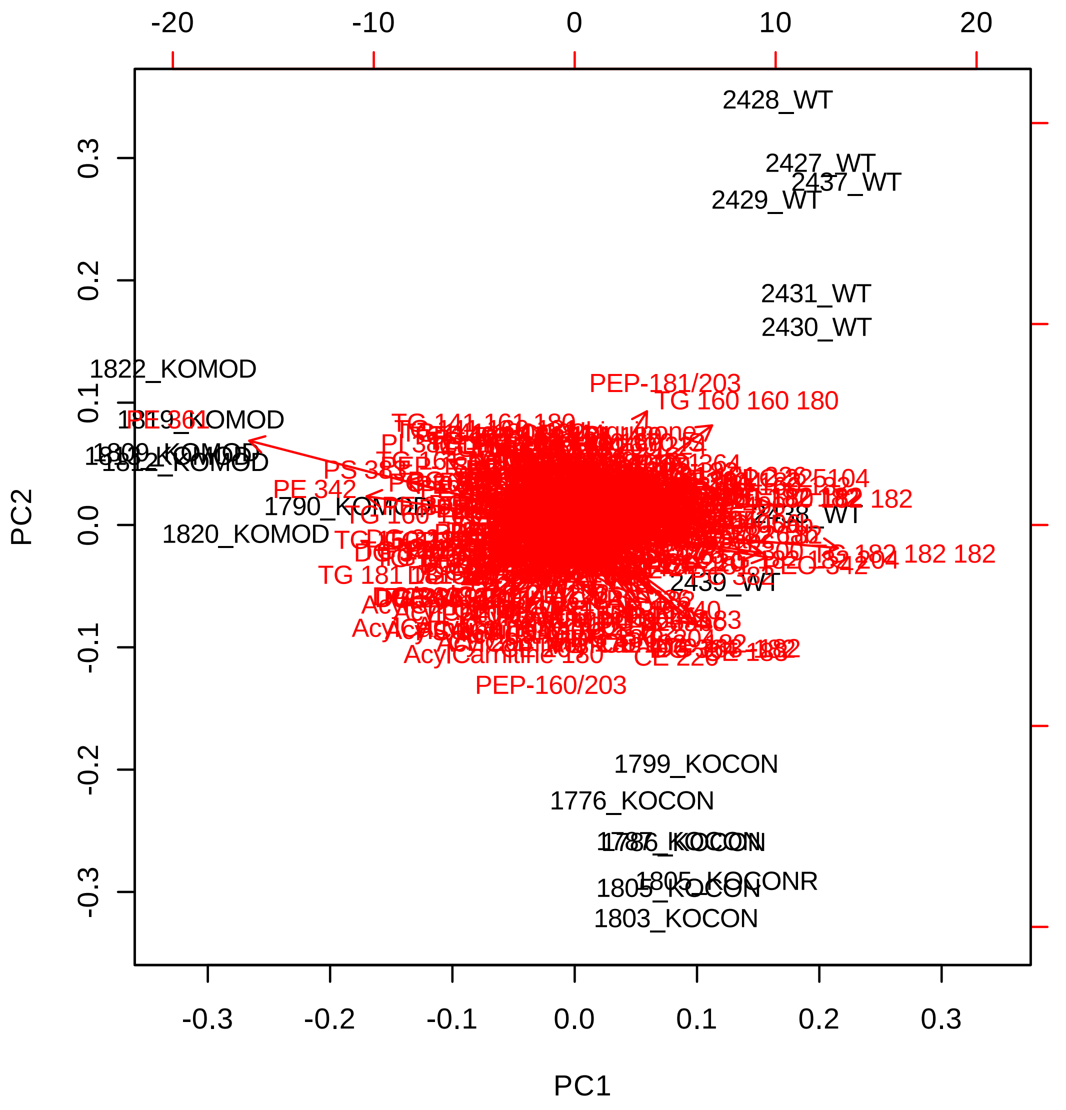

Figure 8: PCA biplot between the selected PCs. Note, you may want to test different centering and scaling normalization methods for the biplot to be displayed properly.

2.3 Hierarchical Clustering

In (agglomerative) hierarchical cluster analysis, each sample begins as a separate cluster and the algorithm proceeds to combine them until all samples belong to one cluster. Two parameters need to be considered when performing hierarchical clustering. The first one is similarity measure - Euclidean distance, Pearson's correlation, Spearman's rank correlation. The other parameter is clustering algorithms, including average linkage (clustering uses the centroids of the observations), complete linkage (clustering uses the farthest pair of observations between the two groups), single linkage (clustering uses the closest pair of observations) and Ward's linkage (clustering to minimize the sum of squares of any two clusters). Heatmap is often presented as a visual aid in addition to the dendrogram.

Hierachical clustering is performed with the `hclust` function in package `stat`. Figure 9 shows the clustering result in the form of a dendrogram. Figure 10 shows the clustering result in the form of a heatmap.

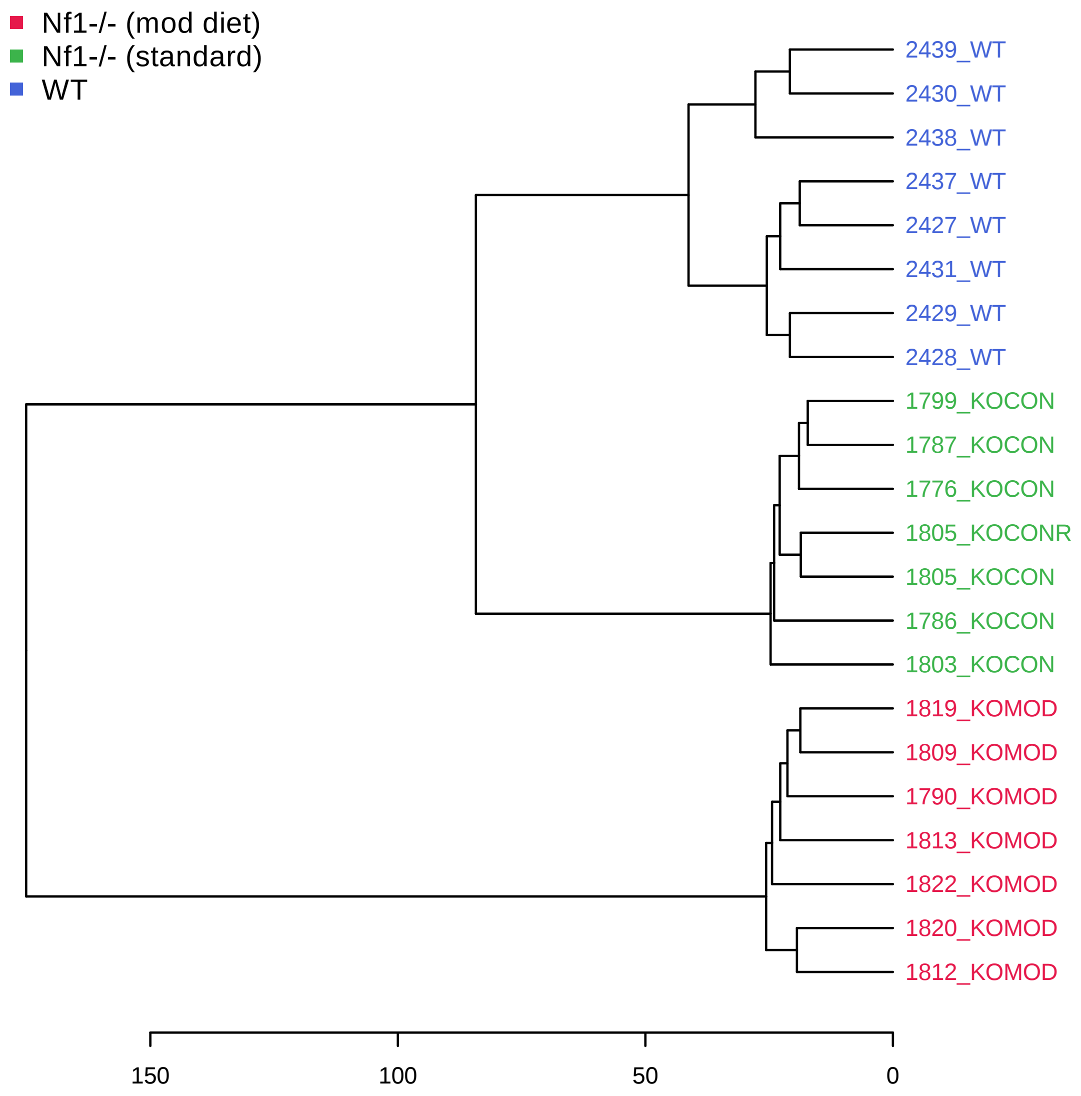

Figure 9: Clustering result shown as dendrogram (distance measure using `euclidean`, and clustering algorithm using `ward.D`).

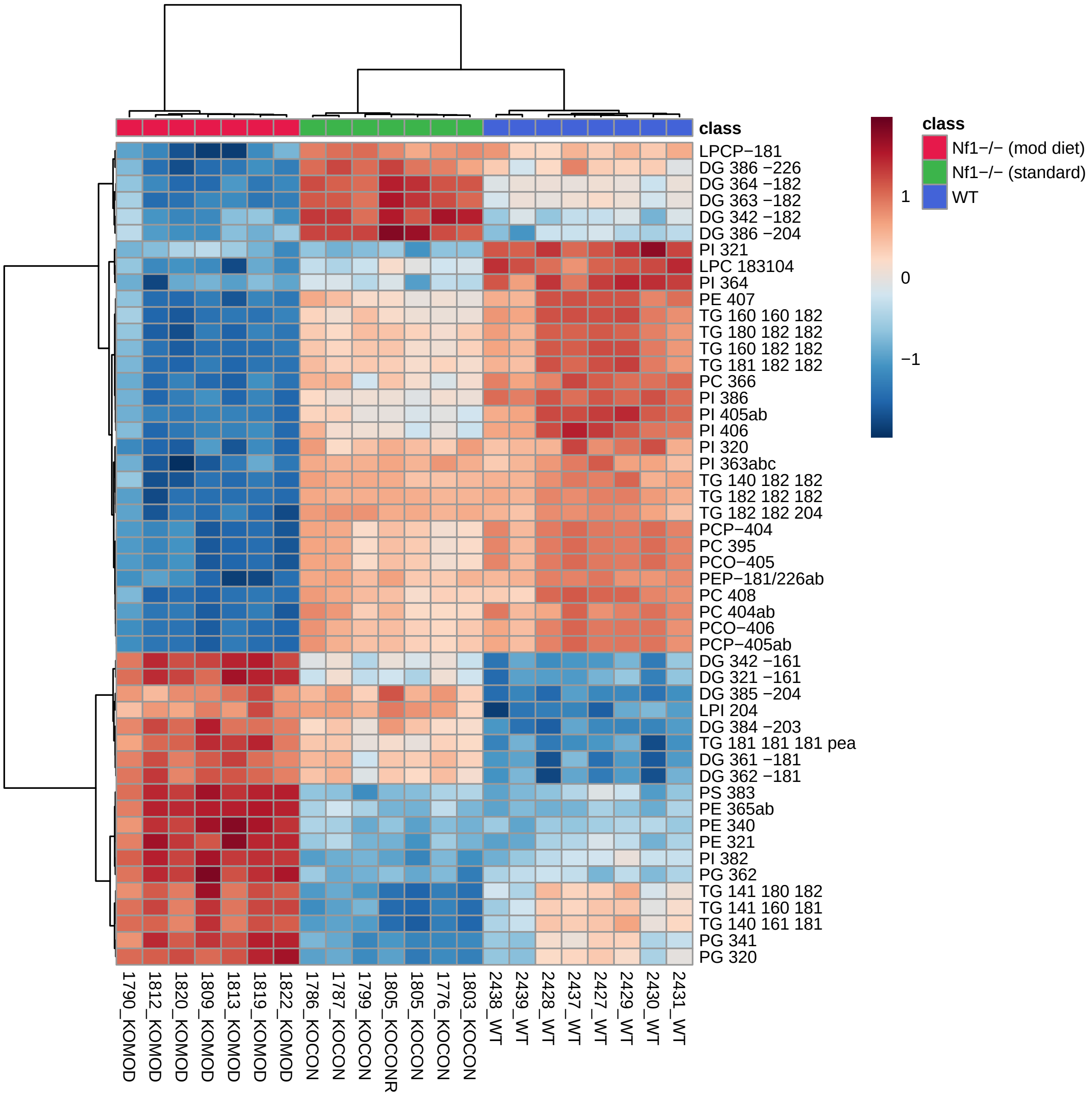

Figure 10: Clustering result shown as heatmap (distance measure using `euclidean`, and clustering algorithm using `ward.D`).

3 Appendix: R Command History

```
 [1] "mSet<-InitDataObjects(\"pktable\", \"stat\", FALSE)"
 [2] "mSet<-Read.TextData(mSet, \"Replacing_with_your_file_path\", \"rowu\", \"disc\");"
 [3] "mSet<-SanityCheckData(mSet)"
 [4] "mSet<-ReplaceMin(mSet);"
 [5] "mSet<-FilterVariable(mSet, \"none\", \"F\", 25)"
 [6] "mSet<-PreparePrenormData(mSet)"
 [7] "mSet<-Normalization(mSet, \"MedianNorm\", \"LogNorm\", \"AutoNorm\", ratio=FALSE, ratioNum=20)
 [8] "mSet<-PlotNormSummary(mSet, \"norm_0_\", \"png\", 72, width=NA)"
 [9] "mSet<-PlotSampleNormSummary(mSet, \"snorm_0_\", \"png\", 72, width=NA)"
[10] "feature.nm.vec <- c(\"\")"
[11] "smpl.nm.vec <- c(\"1792_KOCON\")"
[12] "grp.nm.vec <- c(\"PBQC\")"
[13] "mSet<-UpdateData(mSet)"
[14] "mSet<-PreparePrenormData(mSet)"
[15] "mSet<-Normalization(mSet, \"MedianNorm\", \"LogNorm\", \"AutoNorm\", ratio=FALSE, ratioNum=20)
[16] "mSet<-PlotNormSummary(mSet, \"norm_1_\", \"png\", 72, width=NA)"
[17] "mSet<-PlotSampleNormSummary(mSet, \"snorm_1_\", \"png\", 72, width=NA)"
[18] "mSet<-ANOVA.Anal(mSet, F, 0.05, \"fisher\", FALSE)"
[19] "mSet<-PlotANOVA(mSet, \"aov_0_\", \"png\", 72, width=NA)"
[20] "mSet<-ANOVA.Anal(mSet, F, 0.05, \"fisher\", FALSE)"
[21] "mSet<-PlotANOVA(mSet, \"aov_1_\", \"png\", 72, width=NA)"
[22] "mSet<-PCA.Anal(mSet)"
[23] "mSet<-PlotPCAPairSummary(mSet, \"pca_pair_0_\", \"png\", 72, width=NA, 5)"
[24] "mSet<-PlotPCAScree(mSet, \"pca_scree_0_\", \"png\", 72, width=NA, 5)"
[25] "mSet<-PlotPCA2DScore(mSet, \"pca_score2d_0_\", \"png\", 72, width=NA, 1,2,0.95,0,0)"
[26] "mSet<-PlotPCALoading(mSet, \"pca_loading_0_\", \"png\", 72, width=NA, 1,2);"
[27] "mSet<-PlotPCABiplot(mSet, \"pca_biplot_0_\", \"png\", 72, width=NA, 1,2)"
[28] "mSet<-PlotPCA3DScoreImg(mSet, \"pca_score3d_0_\", \"png\", 72, width=NA, 1,2,3, 40)"
[29] "mSet<-PlotPCA3DLoading(mSet, \"pca_loading3d_0_\", \"json\", 1,2,3)"
[30] "mSet<-PreparePrenormData(mSet)"
[31] "mSet<-Normalization(mSet, \"MedianNorm\", \"LogNorm\", \"NULL\", ratio=FALSE, ratioNum=20)"
[32] "mSet<-PlotNormSummary(mSet, \"norm_2_\", \"png\", 72, width=NA)"
[33] "mSet<-PlotSampleNormSummary(mSet, \"snorm_2_\", \"png\", 72, width=NA)"
[34] "mSet<-PCA.Anal(mSet)"
[35] "mSet<-PlotPCAPairSummary(mSet, \"pca_pair_0_\", \"png\", 72, width=NA, 5)"
[36] "mSet<-PlotPCAScree(mSet, \"pca_scree_0_\", \"png\", 72, width=NA, 5)"
[37] "mSet<-PlotPCA2DScore(mSet, \"pca_score2d_0_\", \"png\", 72, width=NA, 1,2,0.95,0,0)"
[38] "mSet<-PlotPCALoading(mSet, \"pca_loading_0_\", \"png\", 72, width=NA, 1,2);"
[39] "mSet<-PlotPCABiplot(mSet, \"pca_biplot_0_\", \"png\", 72, width=NA, 1,2)"
[40] "mSet<-PlotPCA3DScoreImg(mSet, \"pca_score3d_0_\", \"png\", 72, width=NA, 1,2,3, 40)"
[41] "mSet<-PlotPCA3DLoading(mSet, \"pca_loading3d_0_\", \"json\", 1,2,3)"
[42] "mSet<-ANOVA.Anal(mSet, F, 0.05, \"fisher\", FALSE)"
[43] "mSet<-PlotANOVA(mSet, \"aov_1_\", \"png\", 72, width=NA)"
[44] "mSet<-PlotCmpdView(mSet, \"PI 386\", \"png\", 72, width=NA)"
[45] "mSet<-PlotCmpdView(mSet, \"TG 182 182 182\", \"png\", 72, width=NA)"
[46] "mSet<-PlotCmpdView(mSet, \"TG 182 182 204\", \"png\", 72, width=NA)"
[47] "mSet<-PlotCmpdView(mSet, \"TG 160 160 182\", \"png\", 72, width=NA)"
[48] "mSet<-PlotCmpdView(mSet, \"PI 382\", \"png\", 72, width=NA)"
[49] "mSet<-PlotCmpdView(mSet, \"TG 160 160 182\", \"png\", 72, width=NA)"
[50] "mSet<-PlotCmpdView(mSet, \"TG 181 140 160\", \"png\", 72, width=NA)"
[51] "mSet<-PlotCmpdView(mSet, \"TG 140 182 182\", \"png\", 72, width=NA)"
[52] "mSet<-PlotCmpdView(mSet, \"TG 182 182 204\", \"png\", 72, width=NA)"
[53] "mSet<-PlotCmpdView(mSet, \"TG 180 182 182\", \"png\", 72, width=NA)"
[54] "mSet<-PlotCmpdView(mSet, \"LPCP-181\", \"png\", 72, width=NA)"
[55] "mSet<-PlotCmpdView(mSet, \"DG 386 -204\", \"png\", 72, width=NA)"
[56] "mSet<-PlotCmpdView(mSet, \"DG 364 -182\", \"png\", 72, width=NA)"
```

```
[57] "mSet<-PlotCmpdView(mSet, \"DG 321 -161\", \"png\", 72, width=NA)"
[58] "mSet<-PlotCmpdView(mSet, \"DG 384 -203\", \"png\", 72, width=NA)"
[59] "mSet<-PlotCmpdView(mSet, \"CE 181\", \"png\", 72, width=NA)"
[60] "mSet<-PlotCmpdView(mSet, \"PI 386\", \"png\", 72, width=NA)"
[61] "mSet<-PlotCmpdView(mSet, \"PCO-406\", \"png\", 72, width=NA)"
[62] "mSet<-PlotCmpdView(mSet, \"PC 366\", \"png\", 72, width=NA)"
[63] "mSet<-PlotHCTree(mSet, \"tree_0_\", \"png\", 72, width=NA, \"euclidean\", \"ward.D\")"
[64] "mSet<-PlotHeatMap(mSet, \"heatmap_0_\", \"png\", 72, width=NA, \"norm\", \"row\", \"euclidean\
[65] "mSet<-PlotSubHeatMap(mSet, \"heatmap_1_\", \"png\", 72, width=NA, \"norm\", \"row\", \"euclide
[66] "mSet<-SaveTransformedData(mSet)"
[67] "mSet<-PreparePDFReport(mSet, \"guest8335145726651203987\")\n"
```

The report was generated on Wed Feb 19 05:58:19 2020 with R version 3.6.1 (2019-07-05).
